# Bacterial nitrite production oxidizes Fe(II) bioremediating acidic abandoned coal mine drainage

**DOI:** 10.1128/aem.00405-25

**Published:** 2025-04-16

**Authors:** Anna Vietmeier, Michelle Valkanas, Natalie Lamagna, Samuel Flett, Djuna Gulliver, Nancy Trun

**Affiliations:** 1 Department of Biological Sciences, Duquesne University 189492, Pittsburgh, Pennsylvania, USA; 2 Department of Energy, National Energy Technology Laboratory 17213, Pittsburgh, Pennsylvania, USA; 3 Department of Biology, Earth, and Environmental Science, PennWest California 2130 , California, Pennsylvania, USA; 4 Center for Environmental Research and Education, Duquesne University 6613, Pittsburgh, Pennsylvania, USA; Colorado School of Mines, Golden, Colorado, USA

**Keywords:** bioremediation, biogeochemical cycling, abandoned mine drainage (AMD), passive remediation systems (PRS), iron oxidation, nitrate reduction, nitrate-dependent iron oxidation (NDFO), nitrate reduction-iron oxidation (NRIO)

## Abstract

Passive remediation systems (PRSs) treating either acidic or neutral abandoned coal mine drainage (AMD) are colonized by bacteria that can bioremediate iron (Fe) through chemical cycling. Due to the low pH in acidic AMD, iron oxidation from soluble Fe(II) to precipitated Fe(III) is mainly directed by microbial oxidation. Less well described are biotic reactions that lead to iron remediation through abiotic secondary reactions. We describe here iron oxidation in acidic AMD that is mediated by the bacterial reduction of nitrate to nitrite followed by the geochemical oxidation of Fe(II). Within an acidic PRS, 4,560 bacteria cultured from the microbial community were screened for their ability to oxidize iron and to perform nitrate-dependent iron oxidation (NDFO). Iron oxidation in the culturable community was observed in every pond of the system, ranging from 2.1% to 11.4%, and NDFO was observed in every pond, ranging from 1.4% to 6.0% of the culturable bacteria. Five NDFO isolates were purified and identified as *Paraburkholderia* spp. One of our isolates, *Paraburkholderia* sp. AV18 was shown to drive NDFO through the bacterial production of nitrite that in turn chemically oxidizes Fe(II) (nitrate reduction-iron oxidation; NRIO). AV18 expressed nitrate reductase, *napA*, concurrent to nitrite production. Burkholderiales are found by 16S rRNA gene sequencing in every pond of the PRS. The frequency of NDFO metabolism in the culturable microbial community and abundance of Burkholderiales in the PRS suggest nitrite producers contribute to the bioremediation of iron in acidic AMD and may be an unharnessed opportunity to increase iron bioremediation in acidic conditions.

**IMPORTANCE:**

Our study sheds light on a poorly defined biogeochemical interaction, nitrate-dependent iron oxidation (NDFO), that has been described in several environments. We show that bacterial nitrate reduction produces nitrite, which can chemically oxidize ferrous iron, leading to insoluble ferric iron. We show that bacteria capable of the nitrate reduction-iron oxidation (NRIO) reactions are prevalent throughout multiple passive remediation systems that treat acidic coal mine drainage, indicating this may be a widespread mechanism for iron removal under acidic conditions. In acidic coal mine remediation, iron precipitation has been shown to be solely bacterially mediated, and NRIO provides a simple mechanism for aerobic oxidation of iron in these conditions.

## INTRODUCTION

Abandoned coal mine drainage (AMD) is formed under acidic conditions as a self-propagating process that can last for tens of thousands of years (1–6). AMD can reach the ground surface at either circumneutral (from underground mines in areas with limestone in the soil) or acidic pH (from surface mines). Heavy metals found in AMD persist in the environment as non-biodegradable corrosive agents that decrease microbial soil activity, bioaccumulate in tissues, and are biomagnified along trophic levels (7–9). Heavy metals, like iron, perpetuate total mineral acidity, have undesirable aesthetic effects, and negatively impact human health at elevated levels (7–14).

Passive remediation systems (PRSs) for AMD are composed of various combinations of settling ponds, wetlands, limestone beds, and vertical flow ponds and are specifically designed for either circumneutral AMD or acidic AMD. The goal of PRSs is to increase pH in acidic AMD systems and precipitate metals close to the original contamination source in both acidic and circumneutral PRSs (15). Iron exists in multiple oxidation states; ferrous iron [Fe(II)] is the most common dissolved form of iron, and ferric iron [Fe(III)] is commonly found as a precipitate in circumneutral environments (16–18). Since the PRSs are open to the environment, they are naturally colonized by native microbial communities that impact remediation through their metabolic redox reactions, leading to the biogeochemical cycling of elements (19, 20). In acidic AMD, iron oxidation is bacterial-driven under aerobic, microaerophilic, or anaerobic conditions (17, 21–23).

In addition to direct iron oxidation leading to iron bioremediation in acidic AMD, it could also occur via the cycling of multiple chemicals. Nitrate-dependent iron oxidation (NDFO) has been identified in phylogenetically diverse groups of bacteria in a variety of environments; however, the mechanisms remain obscure (24, 25). The mechanisms used to perform NDFO likely differ under different oxygen levels and pH values. These mechanisms can include biotic-only reactions or biotic–abiotic interactions that rely on enzymes and chemical intermediates (22). The distinction of NDFO mechanisms in different environmental conditions is further elucidated by the ability of acidic NDFO to occur under aerobic conditions as opposed to the strict anaerobic conditions required for neutral NDFO (22, 25, 26).

Our data suggest NDFO could play an important role in the overall effectiveness of bioremediation in acidic AMD. The extent and mechanism of NDFO contribution to microbial iron bioremediation is poorly understood in acidic AMD. Here, we evaluated the extent of direct biological iron oxidation and elucidated the potential for NDFO in the acidic Boyce Park PRS. From screening bacterial isolates in the acidic Boyce Park PRS located in Allegheny County, Pennsylvania, USA, bacteria capable of direct iron oxidation or acidic NDFO were detected in every pond of the system. We determined that the NDFO metabolism in our isolate of *Paraburkholderia* spp. is the result of bacterial nitrate reduction to nitrite that chemically oxidizes Fe(II) in a nitrate reduction-iron oxidation (NRIO) metabolism. Our research elucidates additional pathways that increase iron bioremediation in acidic AMD as well as characterizes the NDFO metabolism in acidic conditions.

## MATERIALS AND METHODS

### Sampling at the acidic Boyce Park PRS and Middle Branch PRS

**Fig 1:**
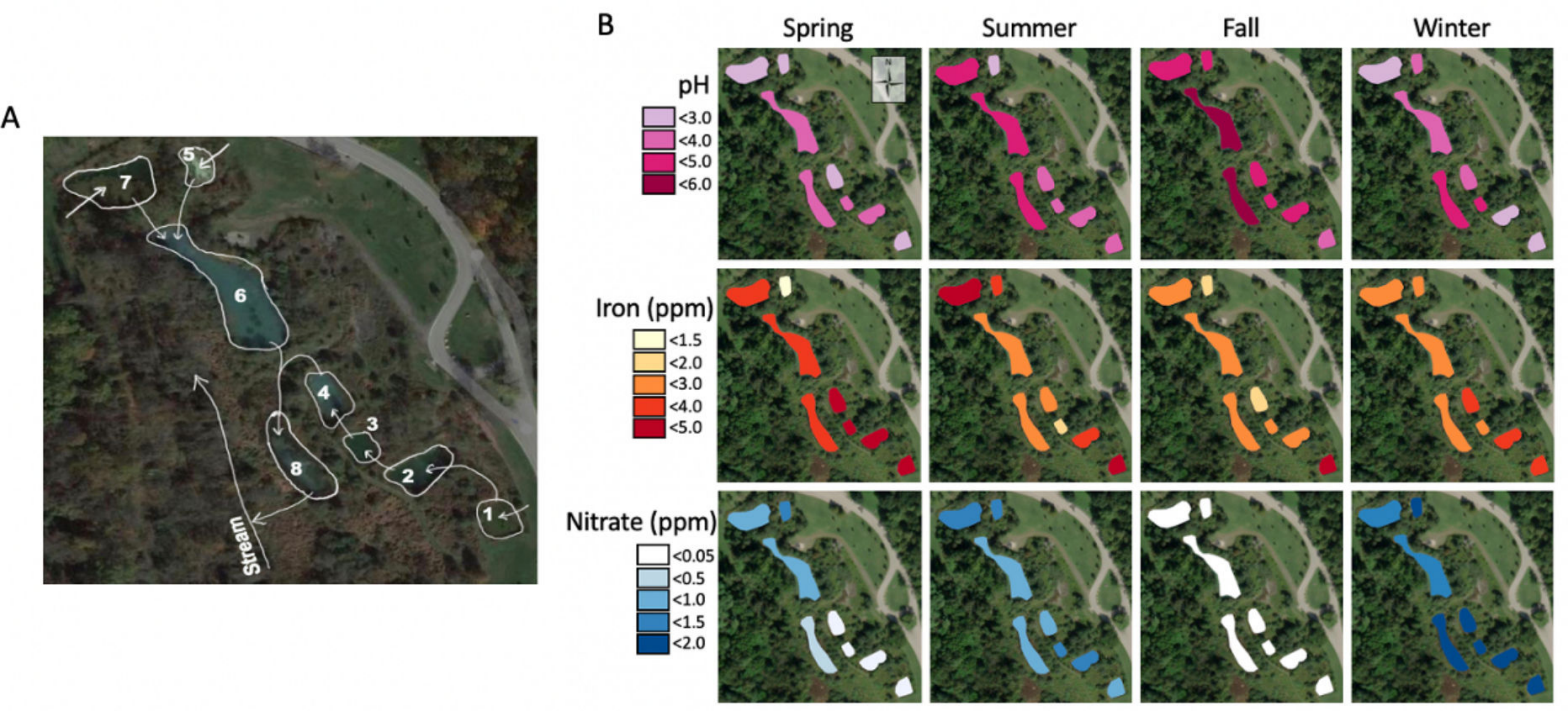
Boyce Park acidic AMD PRS. (A) An aerial image of the Boyce Park PRS was taken from Google Maps to which arrows were added to indicate the flow of AMD through the system. Map data ©2022 Google. (B) Seasonal levels for pH (pink), iron (orange), and nitrate (blue) are indicated.

The Boyce Park PRS was constructed in 2008 with six settling ponds (ponds 1, 2, 3, 4, 6, 7), one limestone bed (pond 5), and a wetland area (pond 8) at latitude 40° 27’ 51.9984” N, longitude 79° 44’ 56.0004” W to treat acidic AMD (Fig. 1A) (27). ArcGIS Pro software was used to visualize our data. The pH data were collected using a YSI Professional Plus Series handheld with YSI Quatro ISE-ISE-DO-COND 18E100032 probe (Xylem Inc., Yellow Spring, OH, USA). Iron in parts per million (PPM) was determined using a PerkinElmer NexION 300x Inductively Coupled Plasma Mass Spectrometry (ICP-MS) with PerkinElmer S10 Autosampler and the NexION 300x ICP-MS software. Nitrate and nitrite in PPM were determined using a Dionex ICS Series ICS-1100 Ultimate 3000 Diode Array Ion Chromatography (IC) (Thermo Fisher Scientific, Waltham, MA, USA) (28). One liter of mud-water slurry samples (containing approximately equal portions of sediment and water from the AMD PRS) was collected from all ponds of the system, a portion of the AMD was autoclaved at 121°C for 45 min, and a non-sterile mixed microbial community sample was maintained for metabolic testing (29). The Middle Branch PRS was constructed in 2001 with two settling ponds (ponds MB1, MB4), four vertical flow ponds (ponds MB2, MB3, MB6, MB7), and wetland area (MB5) ponds, at 41° 20' 48.0012" N, longitude 77° 52' 3" W to treat acidic AMD (pH ~2.8) (Fig. S2) (27, 30).

### Metabolic capabilities of the culturable mixotrophic microbial community for iron oxidation and acidic NDFO in the Boyce Park PRS

To sample the metabolic capabilities of the culturable mixotrophic microbial community for iron oxidation and acidic NDFO, the slurry samples from each pond were diluted and plated for single colonies on R2A agar adjusted to pH 4.0 with HCl, and incubated at 30°C (31). Sterile AMD was confirmed to contain Fe(II) prior to the start of the experiment using the ferrozine assay (32, 33). For the iron oxidation screens, sterile AMD was supplemented with R2A to a final volume of 10%. For the acidic NDFO screens, sterile AMD was supplemented with R2A pH 4.0 to a final volume of 10% and with 5 mM NaNO_3_. The sterile AMD with 10% R2A or the sterile AMD with 10% R2A and 5 mM NaNO_3_ were aliquoted into separate 96-well plates (200 µL per well). Individual colonies from the slurry plates that contained the culturable mixotrophic microbial community were individually inoculated into single wells of sterile 96-well plates with a sterile toothpick. Using one toothpick, a colony was inoculated into a well with 200 µL sterile AMD with 10% R2A to test for iron oxidation, immediately followed by inoculation into a separate single well containing 200 µL sterile AMD with 10% R2A and 5 mM NaNO_3_ to screen for acidic NDFO. The 96-well plates were incubated for 7 days in a closed container with moistened paper towels in the bottom to keep media from drying out in the incubator (wet box). Sterile AMD was used as a negative control to confirm the levels of Fe(II) did not abiotically decrease, and as a qualitative comparison for positive screens for both iron oxidation and acidic NDFO. After incubation, isolates were assessed for iron oxidation and acidic NDFO using the ferrozine assay to detect Fe(II) by the development of a purple color and to detect Fe(II) oxidation by the lack of color development (34). The color development or lack of in each well was enumerated to quantify the metabolic frequency of iron oxidation and acidic NDFO within the culturable mixotrophic community. Five hundred seventy colonies from each pond, for a total of 4,560 bacteria, were screened for iron oxidation and acidic NDFO.

### Identification of NDFO isolates from acidic AMD

Five NDFO bacterial isolates (AV2, AV3, AV18, AV25, and AV26) capable of NDFO were single colony purified twice on R2A agar pH 4.0, and their NDFO phenotypes were confirmed in 10 mL liquid cultures, using the ferrozine assay to measure Fe(II) levels and the Griess assay to observe the production of nitrite (35). Bacterial isolates AV2, AV3, AV18, AV25, and AV26 were recovered from pond 1. DNA was extracted from AV2, AV3, AV18, AV25, and AV26 using the Quick-DNA Fungal/Bacterial Miniprep Plus Kit (Zymo Research, Irvine, CA, USA) following the manufacturer’s instructions. DNA extracted from AV2, AV3, and AV18 was sent for whole genome sequencing at SeqCenter (www.seqcenter.com, SeqCenter, Pittsburgh, PA, USA) using their Illumina MiSeq 200 Mbp package. Sequences received for the forward and reverse reads were imported into KBase (kbase.us) (36). The forward and reverse reads were paired and assembled with SPAdes v.3.13.0, and the quality of the assembly and genome was determined with QUAST v.4.4 (37–41). CheckM v.1.0.18 was used to determine the completeness and contamination of the genome sequence using a set of marker genes (42). Annotation of whole genomes was carried out with Prokka v.1.14.5 (43). The nucleotide Basic Local Alignment Search Tool (BLAST) from the National Center for Biotechnology Information (NCBI) was used to identify AV2, AV3, and AV18 by their 16S rRNA gene nucleic acid sequences (44). Bacterial identity was confirmed by the 97% nucleotide identity match as is standard for bacterial identification (45).

The 16S rRNA gene from AV25 and AV26 were PCR amplified using primers 27F (5´AGAGTTTGATCMTGGCTCAG3´) and 518R (5´GTATTACCGCGGCTGCTGG3´) at 4 mM MgCl_2_ for both AV25 and AV26, and at an annealing temperature of 50°C for AV25 and 60°C for AV26 (46, 47). PCR products were cloned into pCR2.1 and transformed into chemically competent *Escherichia coli* Top10F’ cells using the TOPO TA Cloning Kit and per the manufacturer’s protocol (48). Clones were colony purified three times on Luria-Bertani (LB) plates containing 50 µg/L kanamycin, 40 mg/L X-Gal, and 100 mM isopropyl β-D-thiogalactopyranoside (IPTG). Plasmid DNA was extracted using a ZymoPURE Plasmid Miniprep Kit (Zymo Research, Irvine, CA, USA) following the manufacturer’s instructions. Plasmid DNA was sent for sequencing at GeneWiz (www.genewiz.com, Azenta Life Sciences, South Plainfield, NJ, USA) using Sanger PCR-based sequencing with the GeneWiz M13R primer (5´CAGGAAACAGCTATGAC3´). Sequences were analyzed using 4Peaks v.1.8 (Nucleobytes, The Netherlands) and NCBI BLAST default settings (44).

### Characterization of NRIO

As the uncharacterized NDFO metabolism we observed was in phylogenetically related bacteria, all *Paraburkholderia* spp., the characterization of the NDFO metabolism was carried out in one isolate, AV18. Synthetic AMD was developed based on the average annual chemical levels within the Boyce Park system to minimize seasonal variations between water sampling (Table S1) (4, 49). AV18 was grown for 24 hours in R2A pH 4.0 broth, centrifuged to pellet bacteria, and resuspended in fresh R2A pH 4.0. The cells were diluted to a 1:10 ratio into synthetic AMD. NaNO_3_ was added to a final concentration of 5 mM, and R2A was added to 10%. Samples of the culture were removed periodically (see Results), and the pH of the media was recorded with a Corning Model 440 pH meter 3-in-1 combo probe with RJ pH electrode (Corning Incorporated, Corning, NY, USA). Bacterial growth was measured at OD_600_ concurrently using a Jenway Genova Plus spectrophotometer (Bibby Scientific Ltd., Stone, Staffs, UK). The ferrozine and Griess assays were used to measure Fe and nitrite levels, respectively (32, 33, 35).

The biotic interactions in the NRIO metabolism were determined by the addition of only 5 mM nitrate, only AV18, or both 5 mM nitrate and AV18 to sterile AMD. To rule out direct bacterial iron oxidation, the addition of AV18 to sterile AMD without nitrate was performed. A non-nitrate-reducing organism was included to determine if nitrite production must occur to see NRIO metabolism. AV18 was heat-killed by incubating at 65°C for 2 hours and confirmed to be heat-killed by plating an aliquot on R2A pH 4.0 agar plates concurrent to being added to sterile AMD and amended with nitrate to determine if the cells must be alive and actively growing to reduce nitrate. Chemically driven nitrite iron oxidation was assessed by amending sterile AMD with 1 mM NaNO_2_. To assess bacterially produced nitrite for iron oxidation, AV18 was grown for 24 hours in R2A, was qualitatively tested for the presence of nitrite with the Griess assay, then amended with nitrate and tested for nitrite production. After visual bacterial nitrite production, spent media were filter sterilized with a 0.2 µm filter to remove cells, creating cell-free filtrate containing a metabolically produced NO_2_ byproduct. The sterile filtrate was added into sterilized AMD at 50% and 90% to measure changes in Fe(II). Sterile AMD was used as a control. A two-factor analysis of variance with replication was performed to determine statistical significance using Microsoft Excel v.16.92.

### Design and optimization of novel *napA* and *rpoB* primer sets

Primers specific for AV18 were developed to determine the expression of the periplasmic nitrate reductase, *napA,* during nitrite production. Novel primer sets were developed as *Paraburkholderia* sp. is not a model organism with well-designed *napA* primer sets. This is the first known PCR amplification and target of this specific *napA* gene in this novel organism. KEGG was used to identify potential nitrate reduction genes in the Prokka v.1.14.5 genome annotation of AV18. A novel primer set for the control gene, *rpoB*, was also designed specifically for AV18. Primer sets were designed as nondegenerative with a length of 20–24 base pairs (bp) and a ΔG no less than −6.0 kcal/mol for hairpin loops, self-dimers, and hetero-dimers using the IDT oligo analyzer tool (www.idtdna.com) (Table S2 and S3). Optimal PCR conditions were determined by testing the novel primer sets at 2 mM, 3 mM, and 4 mM MgCl_2_ and at annealing temperatures of 40°C, 50°C, or 60°C for a total of nine conditions using DNA extracted from AV18 as the template. Optimal conditions were determined by single bands of the expected band size on 5% acrylamide gels stained with ethidium bromide and visualized under UV (Fig. S1).

Sanger sequencing of the *napA* PCR product combined with a pairwise alignment to the annotated *napA* nucleotide sequence from the whole genome sequence was used to confirm the novel *napA* primer set (napA_F1 + napA_R1) was targeting the correct gene sequence. The napA_F1 + napA_R1 PCR product was cloned into pCR2.1 and transformed into chemically competent *E. coli* Top10F’ cells with the TOPO TA Cloning Kit and per the manufacturer’s protocol (40). Transformants were selected and purified three times on LB agar with 50 µg/L kanamycin, 40 mg/L X-Gal, and 100 mM IPTG. Plasmid DNA was purified from the clones with the ZymoPURE Plasmid Miniprep Kit (Zymo Research, Irvine, CA, USA) following the manufacturer’s instruction. The DNA sequence of the plasmid inserts was determined by Sanger sequencing using QuantumDye Terminator protocols (QuantumSeq, VH Bio, Gateshead, UK) and the universal M13 forward primer (5´GTAAAAGGACGGCCAG3´). Sanger sequencing was carried out on an ABI 3130 sequencer (Applied Biosystems, Foster City, CA, USA). Sanger sequencing results were visualized with 4Peaks software v.1.8 (Nucleobytes, The Netherlands). The nucleic acid sequence of *napA* was aligned to the annotated whole genome nucleotide sequence using EMBOSS Needle v.6.6.0 to confirm amplification of the target gene was successful.

### Periplasmic nitrate reductase (*napA*) expression during nitrate reduction to nitrite

RNA was extracted from 0.25 mL of 5 mL log phase culture (OD_600_ = 0.500) of AV18 to determine the expression of *napA*. A Griess assay was carried out on a 100 µL portion of the culture to detect the bacterial production of nitrite. Nitrate was added to the culture to a final volume of 1 mM and at 5 min, 30 min, and 120 min post the addition of nitrate, RNA was extracted, and the Griess assay was conducted as above. RNA was extracted using TRIzol (Thermo Fisher Scientific, Waltham, MA, USA). The RNA was reverse transcribed into cDNA using a High-Capacity cDNA Reverse Transcription Kit with RNase Inhibitor (Applied Biosystems, Foster City, CA) according to the manufacturer’s protocol. The cDNA was used with our novel *napA* primers at an annealing temperature of 60°C and 2 mM MgCl_2_ and with our novel *rpoB* primers at an annealing temperature of 60°C and 4 mM MgCl_2_ (50). Reverse transcriptase PCR (RT-PCR) products were visualized under UV light on a 5% acrylamide gel stained with ethidium bromide.

### Presence and abundance of *Paraburkholderia* sp. taxonomic relatives in two acidic AMD PRS at the order, family, and genus level

In a prior study (M. Valkanas, personal communication), two acidic AMD PRS systems (Boyce Park and Middle Branch) were used for a seasonal study to survey the composition of the mixed microbial community using sequences from the 16S rRNA genes in each season (four times per year) in replicates of two within each pond. These sequences can be found under NCBI BioProject accession number PRJNA686480 (Fig. S1). For these sequences, DNA was extracted from slurry samples using the Qiagen DNeasy PowerSoil Kit (Qiagen, Hilden, Germany). The 16S rRNA gene V3 and V4 region was amplified using primers S-D-Bact-0341-b-S-17 (5′CCTACGGGNGGCWGCAG-3′) and S-D-Bact-0785-a-A-21(5′-GACTACHVGGGTATCTAATCC-3) on the Illumina MiSeq following the Illumina 16S Metagenomic Sequencing Library Preparation Protocol (Illumina, San Diego, CA, USA) (51, 52).

In the data reported here, the sequences were downloaded from NCBI and were filtered and analyzed using dada2 v.1.16 in RStudio v.2021.09.01 (53). Analysis with dada2 was carried out for each individual pond within the systems and by pooling the sequences for all four seasons. In dada2, a parametric error model was used to determine the error rate, which was in turn used to dereplicate, filter, and trim the sequence reads. Forward reads had the last 30 bp removed, and reverse reads had the last 20 bp removed. All samples had the first 100 bp removed. Sequence reads were paired to construct an amplicon sequence variant (ASV) matrix that was denoised and had chimeras removed. Taxonomic 16S rRNA gene identity of the AMD PRS mixed bacterial community was assigned to individual ASVs in dada2 using the SILVA v.138.1 data set (54–56). Taxonomic 16S rRNA gene identity text files of the AMD PRS mixed bacterial community generated by dada2 were analyzed in Microsoft Excel v.16.9 to determine total bacterial abundance and abundance of Burkholderiales (order), Burkholderiaceae (family), and *Paraburkholderia-Burkholderia-Caballeronia* (genus) within the community.

## RESULTS

### Seasonal chemical data at Boyce Park PRS for pH, iron, nitrate, and nitrite

The acidic Boyce Park PRS was surveyed to determine its chemical and microbiological profile. Seasonal changes are seen for pH, iron, and nitrate (Fig. 1B). The pH at Boyce Park stays acidic <6.0 during all seasons tested. On average, iron enters the system above the Environmental Protection Agency (EPA) limits for drinking water (0.3 PPM) and freshwater aquatic organism exposure (1.0 PPM) (10, 57–60). Pond 1 of the Boyce Park system is consistently the most heavily contaminated pond within the system, at 3 PPM of iron. Although the amount of detectable iron from pond 1 decreases as it sequentially moves through ponds 2, 3, 4, and finally into pond 8, iron still exits the system above EPA limits at ≥2.0 PPM for aquatic life exposure and drinking water. Overall, the highest levels of iron were detected in the PRS in the spring, excluding pond 5, which was the lowest for all seasons. In fall, the levels of iron and nitrate in the PRS are overall lower compared to the other seasons. Levels of nitrate are below EPA limits for drinking water (44.3 PPM), and nitrite is below detectable levels by IC.

### Frequency of iron oxidation and NDFO within the culturable mixotrophic community in the acidic Boyce Park PRS

**Fig 2:**
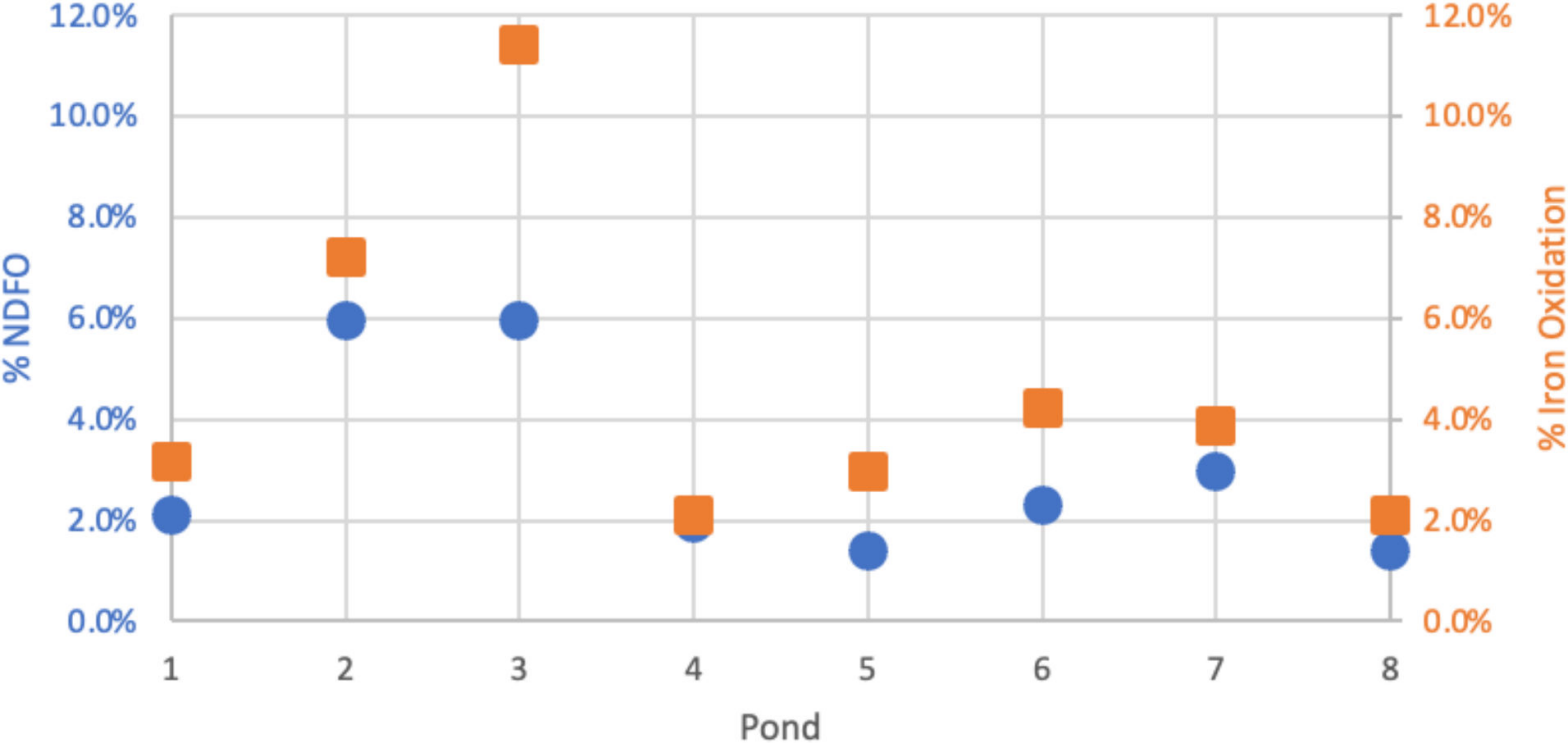
Frequency of iron-oxidizing bacteria (orange square) and acidic NDFO bacteria (blue circle) was determined for all ponds at acidic Boyce Park. The frequency of bacteria with these metabolisms was determined in the culturable mixotrophic bacterial community.

It has been shown that iron oxidation under acidic conditions in a PRS is mediated by bacteria (61). The metabolic frequency of direct iron oxidation and acidic NDFO in bacteria was determined for the culturable mixotrophic bacterial communities for all the ponds at the Boyce Park PRS by screening 570 colonies from each pond, for a total of 4,560 bacteria screened for the entire system (Fig. 2) (62–65). Of the 4,560 culturable bacteria screened, iron oxidation metabolism and acidic NDFO metabolism were found in all ponds at Boyce Park. The lowest frequency of iron oxidation metabolism in the culturable mixotrophic community was found in ponds 4 and 8 at 2.1% (12/570) and the highest frequency of iron oxidation metabolism in pond 3 at 11.4% (65/570). The lowest frequency of NDFO metabolism in the culturable mixotrophic community was found in ponds 5 and 8 at 1.4% (8/570) and the highest frequency of NDFO metabolism in the cultured mixotrophic community in ponds 2 and 3 at 6.0% (34/570).

### Identification of isolates with NDFO phenotypes

Five NDFO isolates from the Boyce Park PRS were purified from NDFO screens to examine the NDFO mechanism. Whole genome sequencing was carried out for bacterial NDFO isolates AV2, AV3, and AV18. These isolates were identified as *Paraburkholderia* spp. with a percent identity of 99%, 100%, and 99%, respectively, to the known *Paraburkholderia acidipaludis* 16S rRNA gene. This is above the 97% identity routinely used for bacterial identification (Table 1) (45). We note that iron oxidase enzyme cytochromes, cyc2 and cyt572, are not present in the annotated whole genome sequence for AV2, AV3, or AV18 (66–69). Isolates AV25 and AV26 were also identified as *Paraburkholderia* sp. via Sanger sequencing of their 16S rRNA gene and their percent identities of 98% to *Paraburkholderia acidipaludis*. As all NDFO isolates identified were *Paraburkholderia* spp., characterization of the NDFO mechanism was carried out in AV18.

**TABLE 1:** Genome characteristics for NDFO isolates AV2, AV3, and AV18

Characteristic	Value for isolate:		
	AV2	AV3	AV18
Genome size	6.74 Mb	6.64 Mb	6.75 Mb
GC content	63%	62%	64%
Number of contigs	126	95	126
Largest contig	366,035 bp	403,425 bp	494,841 bp
Genome completeness	99.82%	99.92%	99.82
Contamination	0.96%	1.34%	0.96%
16S rRNA gene % identity	98.6% *Paraburkholderia* sp.	100% *Paraburkholderia* sp.	98.6% *Paraburkholderia* sp.
Order	Burkholderiales	Burkholderiales	Burkholderiales
Accession number	SAMN40122491	SAMN40122492	SAMN40122493

### Characterization of acidic NDFO as bacterial nitrate reduction chemical iron oxidation

**Fig 3:**
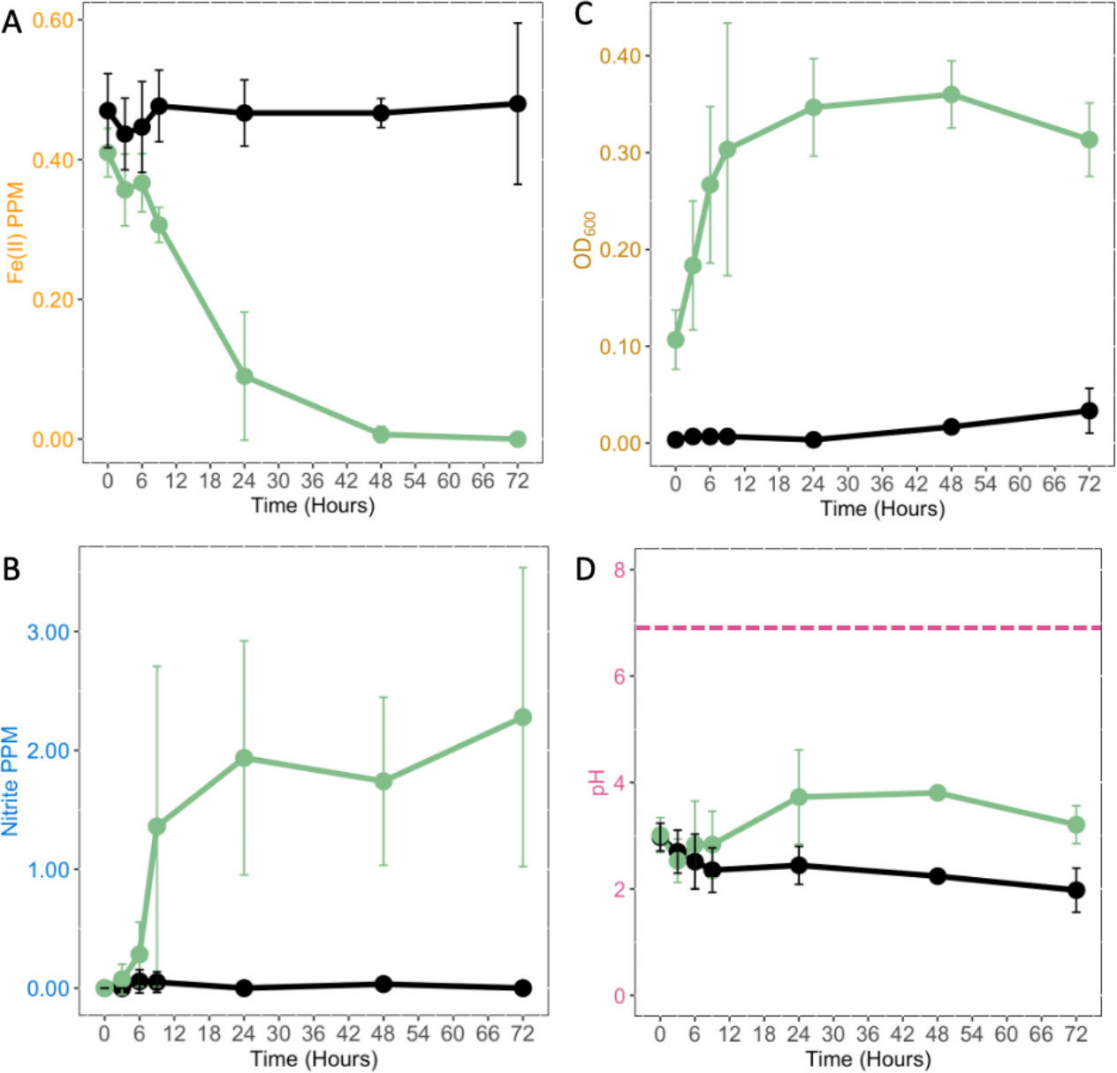
As AV18 grows, it concurrently reduces nitrate and oxidizes iron while the pH remains acidic. Sterile AMD (black line) inoculated with *Paraburkholderia* sp. AV18 (green line) supplemented with 5 mM NaNO_3_ (circle) and 10% R2A results in microbial nitrate reduction to nitrite that chemically oxidizes iron, *n = 3*. (A) Amount of ferrous iron measured in PPM over 72 hours (B) Amount of nitrite measured in PPM over 72 hours. (C) Bacterial growth determined by OD_600_. (D) The pH, the dashed pink line, shows neutral pH. There is a significant difference between samples that remained sterile and those inoculated with *Paraburkholderia* sp. AV18 overtime for Fe(II) (*P* < 0.00001), NO_2_ (*P* = 0.006), and bacterial growth (*P* = 0.002). The pH of the media for both inoculated and sterile AMD remained acidic with no significant change over time (*P* = 0.28).

Characterization of iron oxidation and nitrate reduction in AV18 was quantified in sterile synthetic AMD supplemented with 10% R2A and ±5 mM NaNO_3_ (Fig. 3; Table S1). The decrease in ferrous iron is only observed when 5 mM NaNO_3_ is added to the culture, nitrite is produced, there is concurrent bacterial growth (as measured by OD_600_), and the pH remains acidic. The oxidation of Fe(II) and production of nitrite are only observed when sterile AMD is inoculated with AV18 and supplemented with NaNO_3_ (Fig. 3A and B). As concurrent iron oxidation and nitrite production occur, bacterial growth is detectable, and the pH remains acidic (Fig. 3C and D).

**Fig 4:**
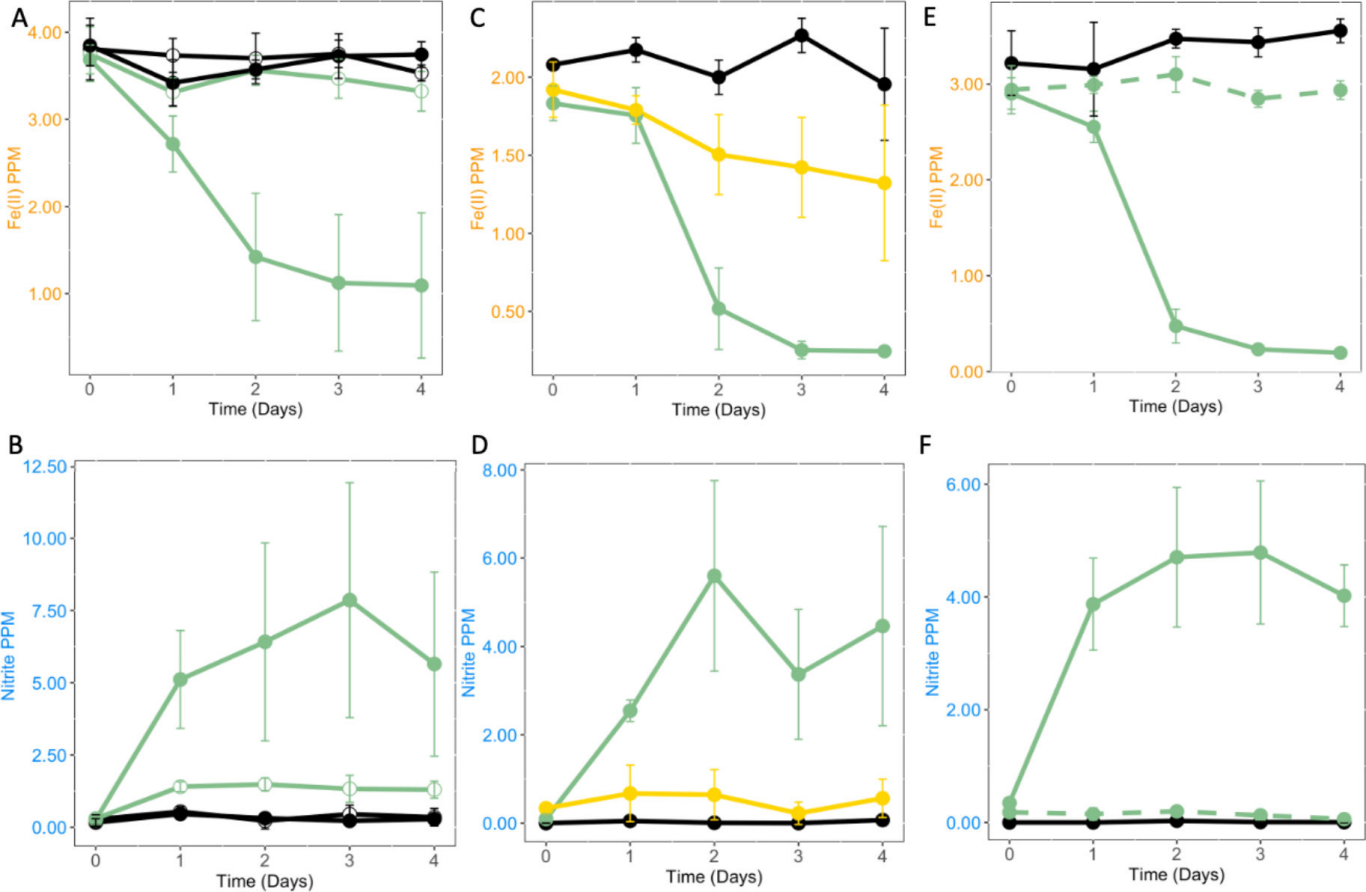
The bacterial-mediated reduction of nitrate to nitrite causes iron oxidation in AMD (*n = 4*). Sterile AMD is shown as a black line, AMD inoculated with *Paraburkholderia* sp. AV18 is shown as a green line, addition of nitrate is a closed circle, no addition of nitrate is an open circle, a non-nitrite-producing isolate is shown as a yellow line, and heat-killed *Paraburkholderia* sp. AV18 is shown by a green dashed line. (A) Iron oxidation is observed when *Paraburkholderia* sp. AV18 is inoculated into sterile AMD and supplemented with nitrate (*P* < 0.000001). Direct bacterial iron oxidation by *Paraburkholderia* sp. AV18 does not occur (*P* = 0.08). (B) Nitrite production is not observed without the addition of both *Paraburkholderia* sp. AV18 and nitrate (*P* = 0.0005). (C) Iron oxidation does not occur to the same extent with the addition of a non-nitrite producer (recovered from the frequency screen) as AMD inoculated with *Paraburkholderia* sp. AV18 (*P* < 0.000001). (D) Nitrate is not reduced to nitrite by the non-nitrite producer (*P* < 0.00001). (E) Iron oxidation does not occur with the addition of heat-killed *Paraburkholderia* sp. AV18 to the same extent as live *Paraburkholderia* sp. AV18 (*P* < 0.00001). The amount of Fe(II) from day 0 to day 4 does not result in a significant change when *Paraburkholderia* sp. AV18 is heat-killed (*P* = 0.33). (F) Nitrite production does not occur when *Paraburkholderia* sp. AV18 is heat-killed before addition to the media (*P* < 0.000001).

Iron oxidation does not occur without the addition of both AV18 and nitrate (Fig. 4A and B). When a non-nitrate-reducing bacterium is used for inoculation, nitrite production does not occur, and iron oxidation is not observed, indicating that the presence of bacterial cells alone does not cause the iron oxidation seen with nitrite producers (Fig. 4C and D). The addition of heat-killed AV18 cells to AMD with 5 mM NaNO_3_ and R2A does not result in the production of nitrite or Fe(II) oxidation (Fig. 4E and F).

**Fig 5:**
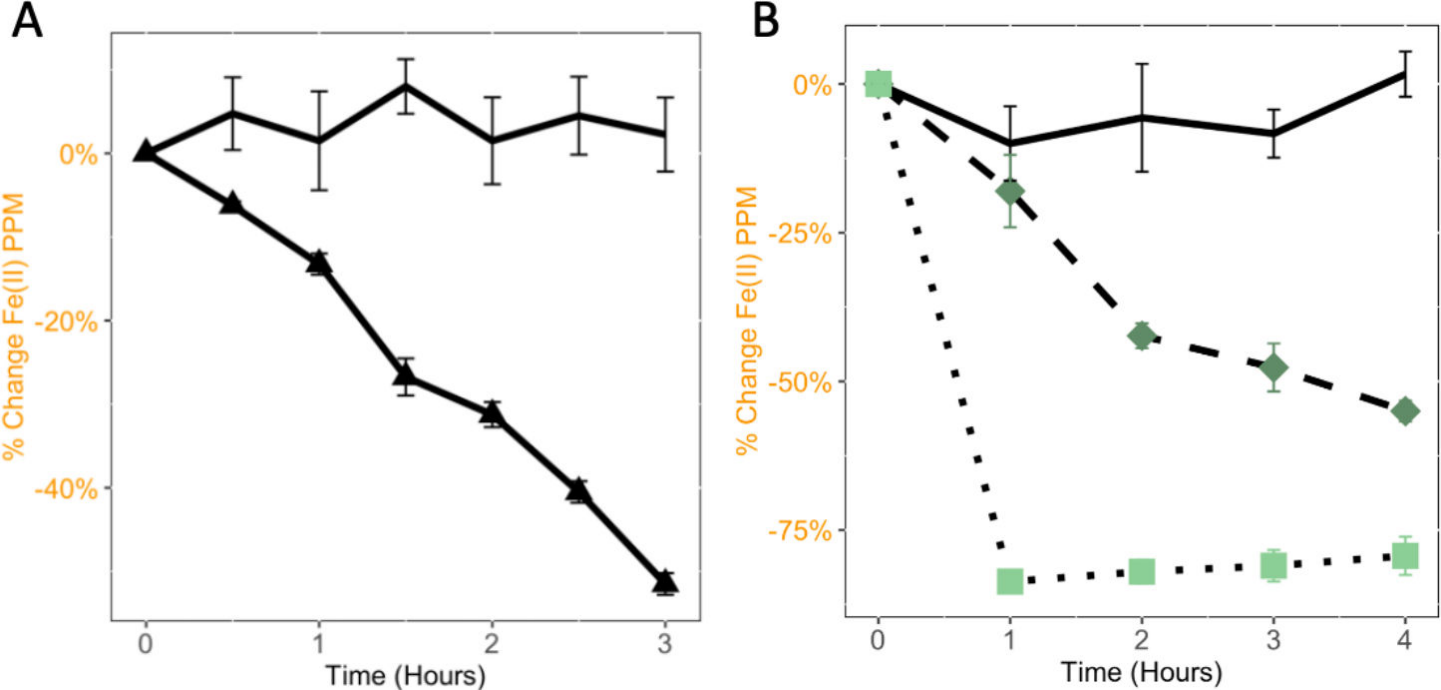
The chemical oxidation of iron by nitrite was determined to occur both with chemical sodium nitrite and bacterial-produced sodium nitrite. Sterile AMD is the black line, AMD amended with laboratory sodium nitrite is shown with a black triangle marker, AMD amended with bacterial *Paraburkholderia* sp. AV18 produced nitrite at 50% is shown with a dark green diamond, and 90% is shown with a light green square. (A) After the addition of laboratory chemical sodium nitrite at time 0 min indicated by the arrow, abiotic chemical Fe(II) oxidation is detected. There was a significant change between sterile AMD with sodium nitrite by hour 3, *n = 4* (*P* < 0.00001). (B) The addition of cell-free sterile nitrite produced by *Paraburkholderia* sp. AV18 to sterile AMD results in detectable iron oxidation. There is a significant difference between sterile AMD and AMD amended with cell-free biotically produced nitrite over time, *n = 3* (*P* < 0.00001).

To determine the extent of abiotic Fe(II) oxidation, chemical grade NaNO_2_ was added to sterile AMD, at a concentration of 1 mM nitrite, and Fe(II) levels were measured (Fig. 5A). Chemical grade NaNO_2_ results in iron oxidation. Nitrite that was produced in an AV18 culture was filtered to remove the bacterial cells and added to AMD at a 1:1 ratio (50%) or a 1:9 ratio (90%) and measured for a decrease in Fe(II) in sterile AMD (Fig. 5B). In all cases of nitrite addition, no matter what the source of nitrite was, iron oxidation was observed.

### *Paraburkholderia* sp. AV18 expresses *napA* during nitrate reduction to nitrite

**Fig 6:**
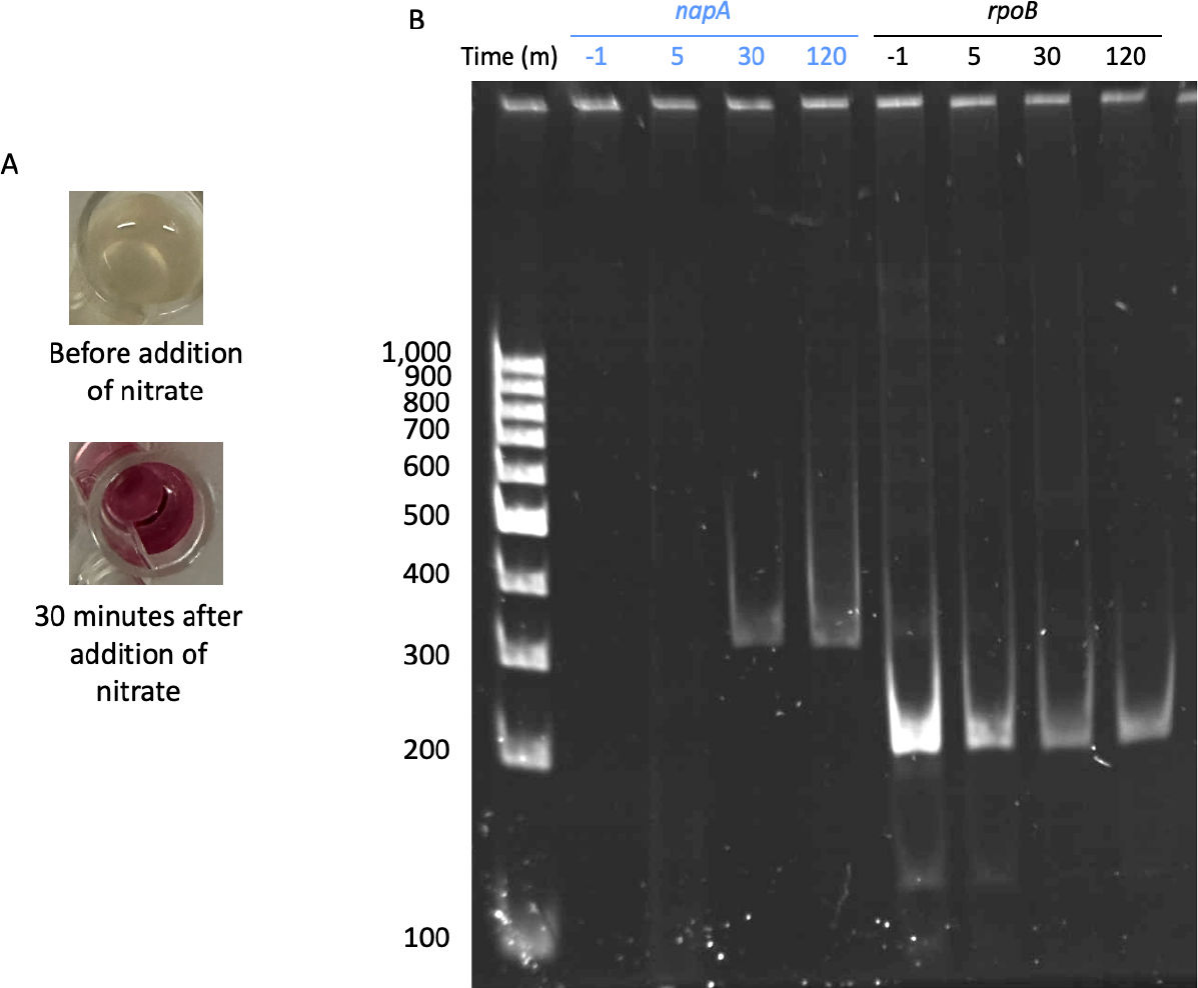
Expression of *napA* occurs when nitrite production by AV18 is observed. (A) Observation of lack of nitrite before the addition of nitrate and the production of nitrite after the addition of nitrate at 30 min. (B) Lane 1 is GeneRuler 100 bp DNA Ladder (Thermo Fisher Scientific, Waltham, MA, USA). Lanes 2–4 *napA* expression, lanes 5–8 *rpoB* control expression.

The whole genome sequence of AV18 predicts a nitrate reductase (*napA*) gene. Primer sets were designed specifically for AV18 *napA*, using *rpoB* as a control. The primers were optimized using chromosomal DNA purified from AV18*,* yielding the predicted sized bands of 352 bp for *napA* and 208 bp for *rpoB* (Fig. S1). The EMBOSS Needle pairwise alignment of the DNA sequence of the *napA* PCR product and the annotated *napA* sequence from the whole genome sequencing resulted in a 100% identity and 100% alignment (352/352 bp). Cultures of AV18 qualitatively assessed using the Griess assay did not contain nitrite before nitrate was added to the media. Bacterially produced nitrite was present in the cultures at 30 min and 120 min (Fig. 6A). RT-PCR using our *napA* and *rpoB* primer sets show that *napA* is expressed by AV18 at 30 min and 120 min (Fig. 6B, lanes 4 and 5). Bands for *rpoB* are present at all time points.

### **Presence and** abundance of Burkholderiales in two acidic AMD PRS Boyce Park and Middle Branch

**Fig 7:**
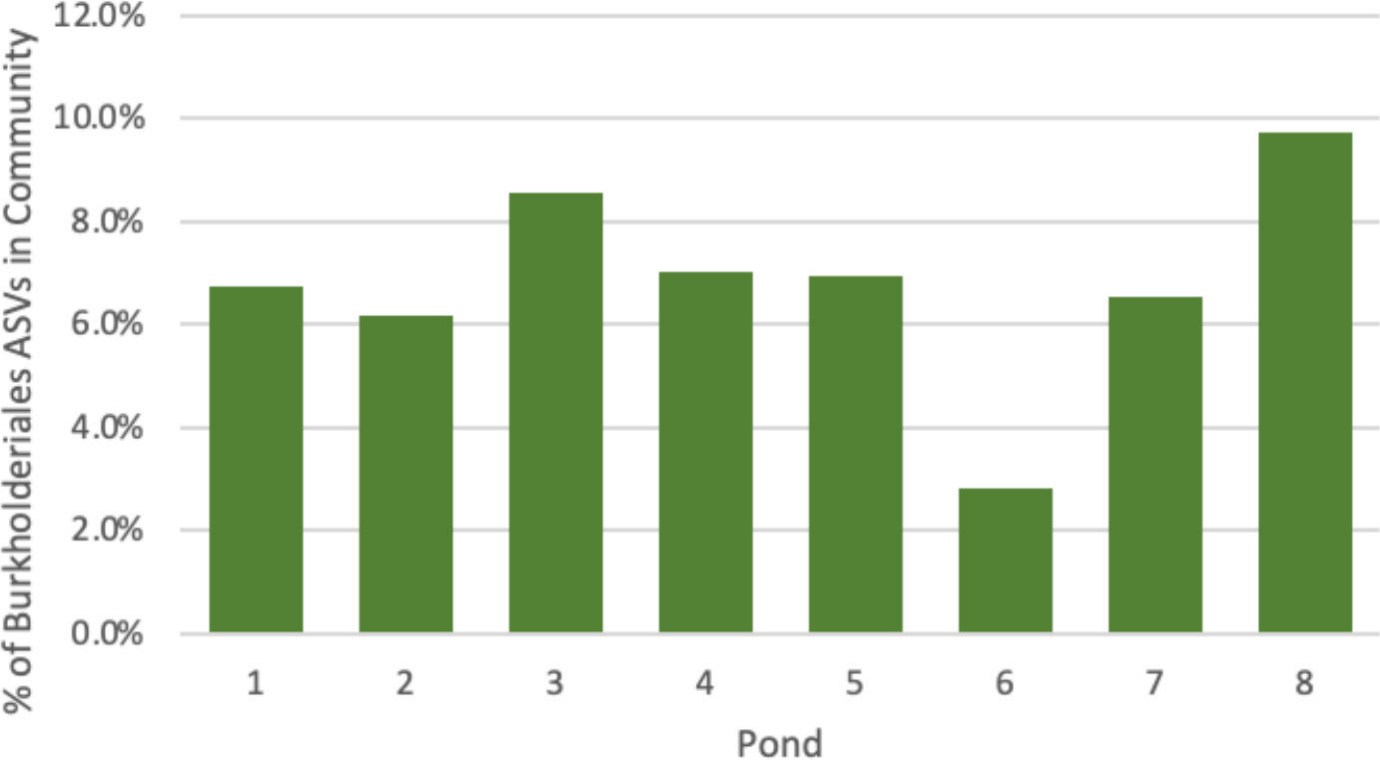
Presence and abundance of Burkholderiales compared to all bacteria within the Boyce Park acidic AMD PRS.

To determine the presence of *Paraburkholderia* sp. phylogenetic relatives in acidic AMD PRS, two PRSs (Boyce Park PRS and Middle Branch PRS) were examined. 16S rRNA gene sequences from our lab for each pond in the system are publicly available (accession number PRJNA686480) (27). The sequences were downloaded from NCBI and mined for phylogenetic relatives to *Paraburkholderia* sp. at the genus (*Paraburkholderia*), family (Burkholderiaceae), and order (Burkholderiales) level. Analysis of the 16S rRNA gene microbial community data from Boyce Park and Middle Branch PRSs was done using the RStudio dada2 pipeline to generate ASV tables that were analyzed in Microsoft Excel v.16.92 (Table S4). Burkholderiales (order level) are predicted in all ponds at Boyce Park and Middle Branch. The abundance of Burkholderiales at Boyce Park ranges from 2.8% to 9.7% (Fig. 7). The abundance of Burkholderiales at Middle Branch PRS ranges from 3.7% to 10.0%. At Boyce, sequencing was able to distinguish Burkholderiaceae in ponds 2, 6, 7, and 8, and *Paraburkholderia-Burkholderia-Caballeronia* were identified in pond 7. At Middle Branch, sequencing was able to distinguish Burkholderiaceae in ponds MB2, MB3, MB4, MB5, and MB6, and *Paraburkholderia-Burkholderia-Caballeronia* were identified in pond MB4.

## DISCUSSION

At a low pH (*≤*6), iron oxidation is mainly microbially mediated as abiotic chemical iron oxidation is not thermodynamically favored under acidic conditions (23, 61). The Boyce Park PRS seasonally stays at a pH <6, and despite levels of detectable iron decreasing from inflow into the system pond 1 at 4 PPM, it still exits the system above EPA limits at ≥2 PPM, leaving room for further system improvements (Fig. 1). Nitrate is detectable by IC at Boyce Park PRS, while nitrite is below detection levels for all seasons, which may be due in part to the rapid chemical interaction of nitrite with Fe(II) (26, 70). Although biogeochemical interactions leading to chemical transformations between microbial-produced nitrite and environmental Fe(II) have been reported in freshwater sediments, coastal marine sediment, and paddy soils, this interaction has been underreported in acidic AMD (17, 22, 24, 70–73). Here, we demonstrate NDFO metabolism can significantly contribute to iron bioremediation and could be further leveraged to optimize bioremediation, reaching discharge goals.

From screening the culturable mixotrophic microbial population at Boyce Park PRS for both iron oxidation and NDFO metabolism, we found bacteria with both metabolisms present throughout the entire system with frequencies between 2.1%–11.4% and 1.4%–6.0%, respectively (Fig. 2). The finding of bacteria in the culturable mixotrophic community that contribute to NDFO in every pond at the Boyce Park system is important because we predict these microbes can be utilized to improve bioremediation. We used this culture-dependent approach of screening 4,569 individual bacterial colonies, in addition to molecular-based culture-independent methods, to better understand and characterize the microbial communities within the Boyce Park PRS (65). In the Boyce Park PRS, 16S rRNA gene sequencing of a total of 7,670 individual ASVs was reported, ranging from 680 to 1,196 ASVs per pond. Direct conclusions and comparisons between the number of microbes detected through molecular approaches and those that are culturable can be challenging to make as it has been estimated that less than 0.1% of microbes are culturable (62–65). Additionally, AMD PRS are generally considered low microbial diversity systems with low species richness and low complexity (3, 74). We do acknowledge the limitations of culture-dependent approaches and the selection bias that can occur based on culturing methods (63, 64). However, NRIO is seen with the *Paraburkholderia* spp. we cultured, and relatives of bacteria are present in both acidic PRS we examined. Future work in these systems can attempt to culture more diverse and novel organisms, including cultivation with iron, nitrate, and NDFO selection media, and with variations of cultivation temperatures (15°C, 22°C, etc.) to better mimic the *in situ* conditions of PRS located within Pennsylvania. There is a need for the cultivation of novel microorganisms from soil environments to better understand their metabolic roles in chemical cycling (65).

Acidic pH NDFO isolates were characterized to elucidate the distinction between neutral pH NDFO and acidic pH NDFO metabolisms (24). We characterized our acidic NDFO metabolism in bacterial isolates under aerobic conditions, in contrast to the NDFO at neutral pH that happens under strict anaerobic conditions (71, 75). Bacterial NDFO isolates AV2, AV3, AV18, AV25, and AV26 were all identified as *Paraburkholderia* sp. by their 16S rRNA gene (Table 1). *Paraburkholderia* sp. is a novel genus that split from Burkholderiales based on distinct *recA* clustering and isolation as nonpathogenic environmental bacteria (76–82). Some *Paraburkholderia* spp. have previously been shown to reduce nitrate to nitrite; iron oxidation has not previously been reported in *Paraburkholderia* spp. (76, 77, 79–84). *Paraburkholderia acidipaulis* was isolated from a Chinese water chestnut that had high sulfur concentrations in highly acidic soils, like the conditions found in AMD (80). The isolation of multiple NRIO *Paraburkholderia* spp. alludes to a strong presence of this bacteria within the PRS.

**Fig 8:**
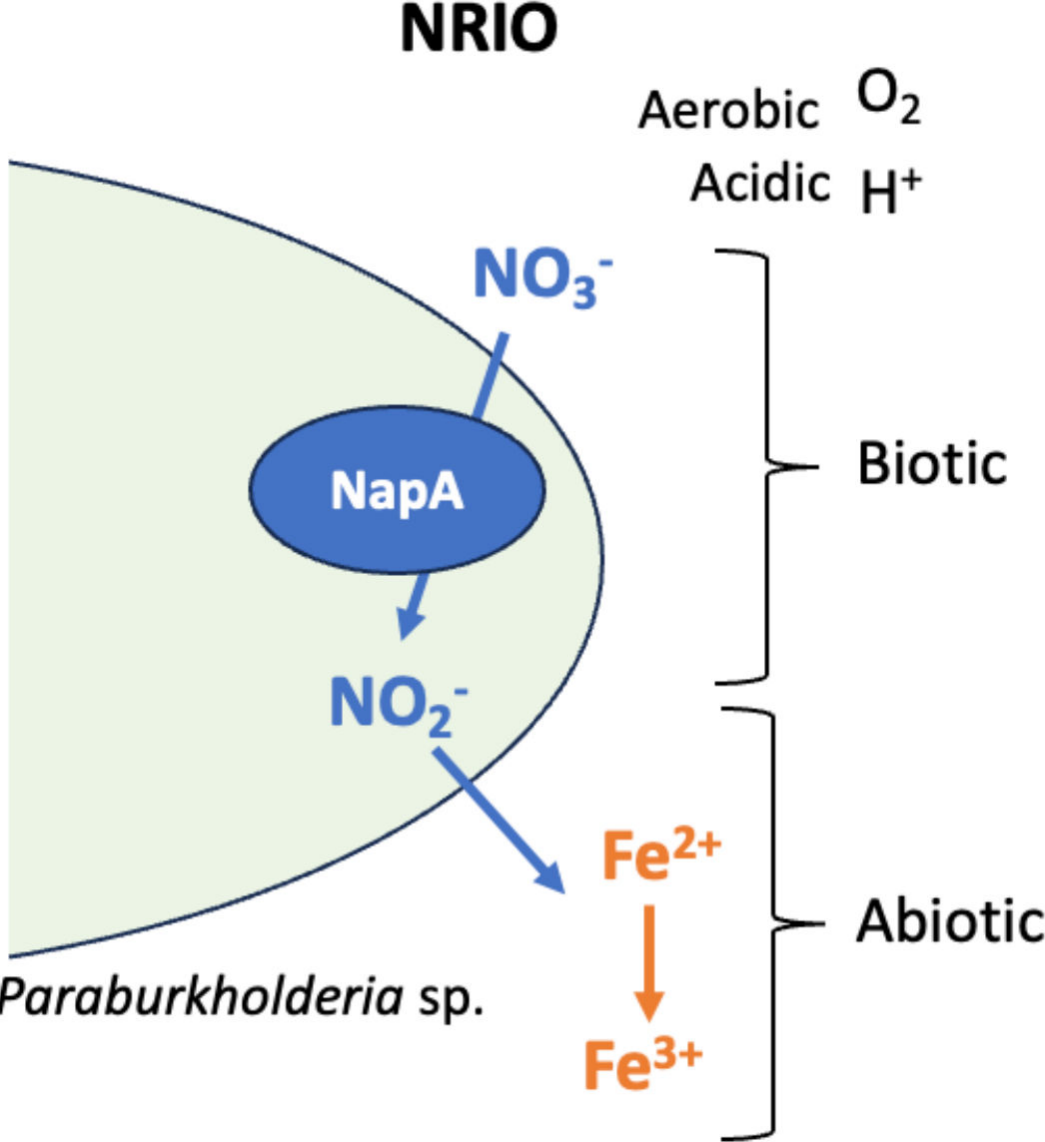
The biotic–abiotic biogeochemical interaction of nitrogen and iron by nitrate-reducing bacteria producing nitrite through NapA leads to chemical iron oxidation in acidic aerobic AMD under laboratory conditions.

We characterized the NDFO mechanism at an acidic pH in AV18 as the biogeochemical interaction of nitrite produced biotically from bacterial nitrate reduction driving chemical Fe(II) oxidation (NRIO metabolism, Fig. 3 and 4 and 5B). AV18 did not directly oxidize iron (Fig. 4A), and its presence alone did not result in iron oxidation (Fig. 4E). Rather, bacteria needed to be able to metabolically produce nitrite from nitrate as the first step. Then the bacterially produced nitrite can oxidize Fe(II) as the second step (Fig. 8). Furthermore, the addition of cell-free bacterially produced nitrite to sterile AMD (Fig. 5B) resulted in iron oxidation as measured by a decrease in Fe(II). In acidic AMD, the addition of nitrite leads to increased iron oxidation, unlike the example of bacterially mediated neutral NDFO reported in the literature (Fig. 5A) (71, 75). In neutral pH environments, bacterially driven NDFO does not include *Paraburkholderia* sp. or *Burkholderia* sp. (22, 24–26, 85). NRIO is most likely enzymatically driven by NapA, as *napA* is expressed during biotic nitrate reduction to nitrite (Fig. 6). Nitrite produced by NapA can react abiotically with dissolved Fe(II), leading to the oxidation and precipitation of Fe(III) (70, 72).

The presence of *Paraburkholderia* spp. phylogenetic relatives at the acidic Boyce Park PRS was determined by mining the microbial community 16S rRNA gene data in an approach to link cultivation results to *in situ* data (Fig. 7). Burkholderiales are present within all ponds of Boyce Park ranging from the lowest in pond 6 at 2.8% (33/1,179 ASVs) and highest in pond 8 at 9.7% (116/1,196 ASVs). At a second acidic PRS, Middle Branch PRS, located at latitude 41° 20' 48.0012" N, longitude 77° 52' 3" W, 65 miles north of state college in Renova, Pennsylvania, was also surveyed for its 16S rRNA community structure following the same methods within this paper (Fig. S1) (27). The AMD in the Middle Branch system is acidic at pH ~2.8 from both surface and underground source mixed together (27, 30). Burkholderiales are present within all ponds at Middle Branch, ranging from the lowest in pond MB3 at 3.7% (30/821 ASVs) and highest in pond MB4 at 10.0% (123/1,234 ASVs) (Fig. S2). The presence of Burkholderiales in two distinct acidic systems (Boyce Park and Middle Branch) alludes to the potential of bioremediation through nitrite production in multiple acidic systems. Additional research will determine if the relatives of *Paraburkholderia* spp. contain the same metabolic capabilities as AV18. We have used a combination of culture-dependent and culture-independent methods to better assess the microbial community and its potential impacts on iron remediation (62, 64).

As nitrate is present within Boyce Park PRS, we hypothesize that nitrate reducers may be an unharnessed microbial strategy to improve iron remediation in acidic AMD passive systems. We conclude that AV18 bioremediates iron by NRIO and suggest that NRIO by *Paraburkholderia* spp. and other nitrate reducers are actively impacting iron removal in acidic AMD (Fig. 8). We propose that promotion of nitrate reduction could be targeted as a strategy to meet discharge levels. AMD PRS can have limited organic matter available, which in turn limits microbial growth as compared to active systems (74). Our work shows the potential of biostimulation of the native nitrate-reducing microbial community could improve iron remediation within an acidic system. We have previously shown the potential for biostimulation of the native microbial community through natural carbon supplementation (86). As nitrate is already present within the system, driving NRIO bacteria to reduce nitrate to nitrite could decrease the concentration of soluble Fe(II) in the system, and may be a faster, more efficient approach for microbial iron bioremediation in acidic AMD or other acidic high iron environments.

## Data Availability

Whole genome sequences were uploaded to NCBI (AV2 accession number SAMN40122491, AV3 accession number SAMN40122492, and AV18 accession number SAMN40122493). Nucleotide sequences of *napA* and *rpoB* from the whole genome sequence have been uploaded to NCBI GenBank under accession numbers BankIt2742455 *napA* OR5574423 and BankIt2742466 *rpoB* OR557424, alongside our novel primer sets for *napA* forward primer napA_F1 (5´AAGAGCGTAAAGAGCGTGTGTCC3´) and reverse primer napA_R1 (5´CGTGCTTGTTGACGAGATACTGCG3´) and *rpoB* forward primer rpoB_F1 (5´CCCATCGTTCACCAGGTTCC3´) and reverse primer rpoB_R1 (5´ATTCCTTGATGTTGAATGCCG3´).

## SUPPLEMENTAL MATERIAL

Supplemental material aem.00405-25-s0001.pdf Tables A1 to A4; Fig. A1 and A2.

ASM does not own the copyrights to Supplemental Material that may be linked to, or accessed through, an article. The authors have granted ASM a non-exclusive, world-wide license to publish the Supplemental Material files. Please contact the corresponding author directly for reuse.

## References

[1] Aguinaga OE, McMahon A, White KN, Dean AP, Pittman JK. 2018. Microbial community shifts in response to acid mine drainage pollution within a natural wetland ecosystem. Front Microbiol 9:1445. doi:10.3389/fmicb.2018.01445 30013541 PMC6036317

[2] Emili LA, Pizarchik J, Mahan CG. 2016. Sustainable remediation of legacy mine drainage: a case study of the flight 93 national memorial. Environ Manage 57:660–670. doi:10.1007/s00267-015-0625-7 26440656

[3] Baker BJ, Banfield JF. 2003. Microbial communities in acid mine drainage. FEMS Microbiol Ecol 44:139–152. doi:10.1016/S0168-6496(03)00028-X 19719632

[4] Muhammad SN, Kusin FM, Zahar MSM, Halimoon N, Yusuf FM. 2015. Passive treatment of acid mine drainage using mixed substrates: batch experiments. Procedia Environ Sci 30:157–161. doi:10.1016/j.proenv.2015.10.028

[5] Dos Santos EC, de Mendonça Silva JC, Duarte HA. 2016. Pyrite oxidation mechanism by oxygen in aqueous medium. J Phys Chem C 120:2760–2768. doi:10.1021/acs.jpcc.5b10949

[6] U.S.E.P.A. 2022. Abandoned mine drainage. Available from: https://www.epa.gov/nps/abandoned-mine-drainage

[7] Kapahi M, Sachdeva S. 2019. Bioremediation options for heavy metal pollution. J Health Pollut 9:191203. doi:10.5696/2156-9614-9.24.191203 31893164 PMC6905138

[8] Kulshreshtha A, Agrawal R, Barar M, Saxena S. 2014. A review on bioremediation of heavy metals in contaminated water. IOSRJESTFT 8:44–50. doi:10.9790/2402-08714450

[9] Singh JK, Yadav KK. 2016. Bioremediation of heavy metals from contaminated sites using potential species: a review. IJEP 37:65–84.

[10] Hallberg KB, Johnson DB. 2005. Biological manganese removal from acid mine drainage in constructed wetlands and prototype bioreactors. Sci Total Environ 338:115–124. doi:10.1016/j.scitotenv.2004.09.011 15680632

[11] Sousa L, Oliveira MM, Pessôa MTC, Barbosa LA. 2020. Iron overload: effects on cellular biochemistry. Clin Chim Acta 504:180–189. doi:10.1016/j.cca.2019.11.029 31790701

[12] Eid R, Arab NTT, Greenwood MT. 2017. Iron mediated toxicity and programmed cell death: a review and a re-examination of existing paradigms. Biochim Biophys Acta Mol Cell Res 1864:399–430. doi:10.1016/j.bbamcr.2016.12.002 27939167

[13] Chow JK, Werner BG, Ruthazer R, Snydman DR. 2010. Increased serum iron levels and infectious complications after liver transplantation. Clin Infect Dis 51:e16–23. doi:10.1086/654802 20578876 PMC2897927

[14] Fonseca-Nunes A, Jakszyn P, Agudo A. 2014. Iron and cancer risk--a systematic review and meta-analysis of the epidemiological evidence. Cancer Epidemiol Biomarkers Prev 23:12–31. doi:10.1158/1055-9965.EPI-13-0733 24243555

[15] Roth H, Gallo S, Badger P, Hillwig M. 2019. Changes in microbial communities of a passive coal mine drainage bioremediation system. Can J Microbiol 65:775–782. doi:10.1139/cjm-2018-0612 31226241

[16] Carter DE. 1995. Oxidation-reduction reactions of metal ions. Environ Health Perspect 103 Suppl 1:17–19. doi:10.1289/ehp.95103s117 PMC1519346 7621791

[17] Bryce C, Blackwell N, Schmidt C, Otte J, Huang Y-M, Kleindienst S, Tomaszewski E, Schad M, Warter V, Peng C, Byrne JM, Kappler A. 2018. Microbial anaerobic Fe(II) oxidation - Ecology, mechanisms and environmental implications. Environ Microbiol 20:3462–3483. doi:10.1111/1462-2920.14328 30058270

[18] Azzam AM, Elatrash AM, Ghattas NK. 1969. The co-precipitation of manganese by iron(III) hydroxide. J Radioanal Chem 2:255–262. doi:10.1007/BF02513737

[19] Tebo BM, Johnson HA, McCarthy JK, Templeton AS. 2005. Geomicrobiology of manganese(II) oxidation. Trends Microbiol 13:421–428. doi:10.1016/j.tim.2005.07.009 16054815

[20] Kracke F, Vassilev I, Krömer JO. 2015. Microbial electron transport and energy conservation - the foundation for optimizing bioelectrochemical systems. Front Microbiol 6:575. doi:10.3389/fmicb.2015.00575 26124754 PMC4463002

[21] Kappler A, Straub KL. 2005. Geomicrobiological cycling of iron. Rev Mineral Geochem 59:85–108. doi:10.2138/rmg.2005.59.5

[22] Carlson HK, Clark IC, Melnyk RA, Coates JD. 2012. Toward a mechanistic understanding of anaerobic nitrate-dependent iron oxidation: balancing electron uptake and detoxification. Front Microbiol 3:57. doi:10.3389/fmicb.2012.00057 22363331 PMC3282478

[23] Singer PC, Stumm W. 1970. Acidic mine drainage: the rate-determining step. Science 167:1121–1123. doi:10.1126/science.167.3921.1121 17829406

[24] Picardal F. 2012. Abiotic and microbial interactions during anaerobic transformations of Fe(II) and [formula: see text]. Front Microbiol 3:112. doi:10.3389/fmicb.2012.00112 22479259 PMC3314871

[25] Chakraborty A, Picardal F. 2013. Induction of nitrate-dependent Fe(II) oxidation by Fe(II) in Dechloromonas sp. strain UWNR4 and Acidovorax sp. strain 2AN. Appl Environ Microbiol 79:748–752. doi:10.1128/AEM.02709-12 23144134 PMC3553780

[26] Beller HR, Zhou P, Legler TC, Chakicherla A, Kane S, Letain TE, A O’Day P. 2013. Genome-enabled studies of anaerobic, nitrate-dependent iron oxidation in the chemolithoautotrophic bacterium Thiobacillus denitrificans. Front Microbiol 4:249. doi:10.3389/fmicb.2013.00249 24065960 PMC3753534

[27] Valkanas M. 2020. Identifying the effects naturally forming bacterial communities have on the efficiency of passive remediation systems built to treat abandoned coal mine drainage, p 532. In Biological sciences. Duquesne University.

[28] Cantlay T, Bain DJ, Curet J, Jack RF, Dickson BC, Basu P, Stolz JF. 2020. Determining conventional and unconventional oil and gas well brines in natural sample II: cation analyses with ICP-MS and ICP-OES. J Environ Sci Health A Tox Hazard Subst Environ Eng 55:11–23. doi:10.1080/10934529.2019.1666561 31549915

[29] Valkanas MM, Trun NJ. 2018. A seasonal study of a passive abandoned coalmine drainage remediation system reveals three distinct zones of contaminant levels and microbial communities. Microbiologyopen 7:e00585. doi:10.1002/mbo3.585 29696823 PMC6079175

[30] Datashed, middle branch - two mile run. 2024. Available from: https://datashed.com

[31] Reasoner DJ, Geldreich EE. 1985. A new medium for the enumeration and subculture of bacteria from potable water. Appl Environ Microbiol 49:1–7. doi:10.1128/aem.49.1.1-7.1985 3883894 PMC238333

[32] Stookey LL. 1970. Ferrozine---a new spectrophotometric reagent for iron. Anal Chem 42:779–781. doi:10.1021/ac60289a016

[33] Viollier E, Inglett PW, Hunter K, Roychoudhury AN, Van Cappellen P. 2000. The ferrozine method revisited: Fe(II)/Fe(III) determination in natural waters. Appl Geochem 15:785–790. doi:10.1016/S0883-2927(99)00097-9

[34] Verschoor MJ, Molot LA. 2013. A comparison of three colorimetric methods of ferrous and total reactive iron measurement in freshwaters. Limnol Ocean Method 11:113–125. doi:10.4319/lom.2013.11.113

[35] Granger DL, Taintor RR, Boockvar KS, Hibbs Jr JB. 1995. Determination of nitrate and nitrite in biological samples using bacterial nitrate reductase coupled with griess reaction. Methods 7:78–83. doi:10.1006/meth.1995.1011 8782580

[36] Arkin AP, Cottingham RW, Henry CS, Harris NL, Stevens RL, Maslov S, Dehal P, Ware D, Perez F, Canon S, et al.. 2018. KBase: the united states department of energy systems biology knowledgebase. Nat Biotechnol 36:566–569. doi:10.1038/nbt.4163 29979655 PMC6870991

[37] Bankevich A, Nurk S, Antipov D, Gurevich AA, Dvorkin M, Kulikov AS, Lesin VM, Nikolenko SI, Pham S, Prjibelski AD, Pyshkin AV, Sirotkin AV, Vyahhi N, Tesler G, Alekseyev MA, Pevzner PA. 2012. SPAdes: a new genome assembly algorithm and its applications to single-cell sequencing. J Comput Biol 19:455–477. doi:10.1089/cmb.2012.0021 22506599 PMC3342519

[38] Prjibelski A, Antipov D, Meleshko D, Lapidus A, Korobeynikov A. 2020. Using SPAdes de novo assembler. Curr Protoc Bioinformatics 70:e102. doi:10.1002/cpbi.102 32559359

[39] Gurevich A, Saveliev V, Vyahhi N, Tesler G. 2013. QUAST: quality assessment tool for genome assemblies. Bioinformatics 29:1072–1075. doi:10.1093/bioinformatics/btt086 23422339 PMC3624806

[40] Mikheenko A, Valin G, Prjibelski A, Saveliev V, Gurevich A. 2016. Icarus: visualizer for de novo assembly evaluation. Bioinformatics 32:3321–3323. doi:10.1093/bioinformatics/btw379 27378299

[41] Bioinformatics Group at the Babraham Institute, U. 2024. A quality control tool for high throughput sequence data. Available from: http://www.bioinformatics.babraham.ac.uk/projects/fastqc

[42] Parks DH, Imelfort M, Skennerton CT, Hugenholtz P, Tyson GW. 2015. CheckM: assessing the quality of microbial genomes recovered from isolates, single cells, and metagenomes. Genome Res 25:1043–1055. doi:10.1101/gr.186072.114 25977477 PMC4484387

[43] Seemann T. 2014. Prokka: rapid prokaryotic genome annotation. Bioinformatics 30:2068–2069. doi:10.1093/bioinformatics/btu153 24642063

[44] Altschul SF, Gish W, Miller W, Myers EW, Lipman DJ. 1990. Basic local alignment search tool. J Mol Biol 215:403–410. doi:10.1016/S0022-2836(05)80360-2 2231712

[45] Stackebrandt E, Goebel BM. 1994. Taxonomic note: a place for DNA-DNA reassociation and 16S rRNA sequence analysis in the present species definition in bacteriology. Int J Syst Evol Microbiol 44:846–849. doi:10.1099/00207713-44-4-846

[46] Goolam Mahomed T, Peters R, Pretorius G, Goolam Mahomed A, Ueckermann V, Kock MM, Ehlers MM. 2021. Comparison of targeted metagenomics and IS-Pro methods for analysing the lung microbiome. BMC Microbiol 21:228. doi:10.1186/s12866-021-02288-x 34407769 PMC8371770

[47] Das S, Dash HR, Mangwani N, Chakraborty J, Kumari S. 2014. Understanding molecular identification and polyphasic taxonomic approaches for genetic relatedness and phylogenetic relationships of microorganisms. J Microbiol Methods 103:80–100. doi:10.1016/j.mimet.2014.05.013 24886836

[48] Liu J, Lee W, Jiang Z, Chen Z, Jhunjhunwala S, Haverty PM, Gnad F, Guan Y, Gilbert HN, Stinson J, et al.. 2012. Genome and transcriptome sequencing of lung cancers reveal diverse mutational and splicing events. Genome Res 22:2315–2327. doi:10.1101/gr.140988.112 23033341 PMC3514662

[49] Senko JM, Wanjugi P, Lucas M, Bruns MA, Burgos WD. 2008. Characterization of Fe(II) oxidizing bacterial activities and communities at two acidic Appalachian coalmine drainage-impacted sites. ISME J 2:1134–1145. doi:10.1038/ismej.2008.60 18548117

[50] Henson MA, Roberts AC, Salimi K, Vadlamudi S, Hamer RM, Gilmore JH, Jarskog LF, Philpot BD. 2008. Developmental regulation of the NMDA receptor subunits, NR3A and NR1, in human prefrontal cortex. Cereb Cortex 18:2560–2573. doi:10.1093/cercor/bhn017 18296432 PMC2733318

[51] Herlemann DP, Labrenz M, Jürgens K, Bertilsson S, Waniek JJ, Andersson AF. 2011. Transitions in bacterial communities along the 2000 km salinity gradient of the Baltic Sea. ISME J 5:1571–1579. doi:10.1038/ismej.2011.41 21472016 PMC3176514

[52] Klindworth A, Pruesse E, Schweer T, Peplies J, Quast C, Horn M, Glöckner FO. 2013. Evaluation of general 16S ribosomal RNA gene PCR primers for classical and next-generation sequencing-based diversity studies. Nucleic Acids Res 41:e1. doi:10.1093/nar/gks808 22933715 PMC3592464

[53] Callahan BJ, McMurdie PJ, Rosen MJ, Han AW, Johnson AJA, Holmes SP. 2016. DADA2: high-resolution sample inference from Illumina amplicon data. Nat Methods 13:581–583. doi:10.1038/nmeth.3869 27214047 PMC4927377

[54] Quast C, Pruesse E, Yilmaz P, Gerken J, Schweer T, Yarza P, Peplies J, Glöckner FO. 2013. The SILVA ribosomal RNA gene database project: improved data processing and web-based tools. Nucleic Acids Res 41:D590–6. doi:10.1093/nar/gks1219 23193283 PMC3531112

[55] Yilmaz P, Parfrey LW, Yarza P, Gerken J, Pruesse E, Quast C, Schweer T, Peplies J, Ludwig W, Glöckner FO. 2014. The SILVA and “all-species living tree project (LTP)” taxonomic frameworks. Nucleic Acids Res 42:D643–8. doi:10.1093/nar/gkt1209 24293649 PMC3965112

[56] Glöckner FO, Yilmaz P, Quast C, Gerken J, Beccati A, Ciuprina A, Bruns G, Yarza P, Peplies J, Westram R, Ludwig W. 2017. 25 years of serving the community with ribosomal RNA gene reference databases and tools. J Biotechnol 261:169–176. doi:10.1016/j.jbiotec.2017.06.1198 28648396

[57] Cravotta CA. 2008. Dissolved metals and associated constituents in abandoned coal-mine discharges, Pennsylvania, USA. Part 1: Constituent quantities and correlations. Appl Geochem 23:166–202. doi:10.1016/j.apgeochem.2007.10.011

[58] EPAUSEPA. 2022 Drinking water regulations and contaminants. Available from: https://www.epa.gov/sdwa/drinking-water-regulations-and-contaminants

[59] EPA, U.S.E.P.A. 2022. National recommended water quality criteria - aquatic life criteria table. Available from: https://www.epa.gov/wqc/national-recommended-water-quality-criteria-aquatic-life-criteria-table

[60] Hatat-Fraile MM, Barbeau B. 2019. Performance of colorimetric methods for the analysis of low levels of manganese in water. Talanta 194:786–794. doi:10.1016/j.talanta.2018.11.003 30609606

[61] Bird LJ, Bonnefoy V, Newman DK. 2011. Bioenergetic challenges of microbial iron metabolisms. Trends Microbiol 19:330–340. doi:10.1016/j.tim.2011.05.001 21664821

[62] Lagkouvardos I, Overmann J, Clavel T. 2017. Cultured microbes represent a substantial fraction of the human and mouse gut microbiota. Gut Microbes 8:493–503. doi:10.1080/19490976.2017.1320468 28418756 PMC5628658

[63] Hugenholtz P, Goebel BM, Pace NR. 1998. Impact of culture-independent studies on the emerging phylogenetic view of bacterial diversity. J Bacteriol 180:4765–4774. doi:10.1128/JB.180.18.4765-4774.1998 9733676 PMC107498

[64] Amann RI, Ludwig W, Schleifer KH. 1995. Phylogenetic identification and in situ detection of individual microbial cells without cultivation. Microbiol Rev 59:143–169. doi:10.1128/mr.59.1.143-169.1995 7535888 PMC239358

[65] Hill GT, Mitkowski NA, Aldrich-Wolfe L, Emele LR, Jurkonie DD, Ficke A, Maldonado-Ramirez S, Lynch ST, Nelson EB. 2000. Methods for assessing the composition and diversity of soil microbial communities. Agric, Ecosyst Environ, Appl Soil Ecol 15:25–36. doi:10.1016/S0929-1393(00)00069-X

[66] Jeans C, Singer SW, Chan CS, Verberkmoes NC, Shah M, Hettich RL, Banfield JF, Thelen MP. 2008. Cytochrome 572 is a conspicuous membrane protein with iron oxidation activity purified directly from a natural acidophilic microbial community. ISME J 2:542–550. doi:10.1038/ismej.2008.17 18463612

[67] Chan CS, McAllister SM, Garber AI, Hallahan BJ, Rozovsky S. 2018. Fe oxidation by a fused cytochrome-porin common to diverse Fe-oxidizing bacteria. Biorxiv. doi:10.1101/228056 PMC8406198 34311573

[68] Keffer JL, McAllister SM, Garber AI, Hallahan BJ, Sutherland MC, Rozovsky S, Chan CS. 2021. Iron oxidation by a fused cytochrome-porin common to diverse iron-oxidizing bacteria. MBio 12:e0107421. doi:10.1128/mBio.01074-21 34311573 PMC8406198

[69] McAllister SM, Vandzura R, Keffer JL, Polson SW, Chan CS. 2021. Aerobic and anaerobic iron oxidizers together drive denitrification and carbon cycling at marine iron-rich hydrothermal vents. ISME J 15:1271–1286. doi:10.1038/s41396-020-00849-y 33328652 PMC8114936

[70] Klueglein N, Zeitvogel F, Stierhof Y-D, Floetenmeyer M, Konhauser KO, Kappler A, Obst M. 2014. Potential role of nitrite for abiotic Fe(II) oxidation and cell encrustation during nitrate reduction by denitrifying bacteria. Appl Environ Microbiol 80:1051–1061. doi:10.1128/AEM.03277-13 24271182 PMC3911208

[71] Li M-J, Wei M-Y, Fan X-T, Zhou G-W. 2022. Underestimation about the contribution of nitrate reducers to iron cycling indicated by Enterobacter strain. Molecules 27:17. doi:10.3390/molecules27175581 PMC9457790 36080348

[72] Li S, Li X, Li F. 2018. Fe(II) oxidation and nitrate reduction by a denitrifying bacterium, Pseudomonas stutzeri LS-2, isolated from paddy soil. J Soils Sediments 18:1668–1678. doi:10.1007/s11368-017-1883-1

[73] Chen D, Yuan X, Zhao W, Luo X, Li F, Liu T. 2020. Chemodenitrification by Fe(II) and nitrite: pH effect, mineralization and kinetic modeling. Chem Geol 541:119586. doi:10.1016/j.chemgeo.2020.119586

[74] Méndez-García C, Peláez AI, Mesa V, Sánchez J, Golyshina OV, Ferrer M. 2015. Microbial diversity and metabolic networks in acid mine drainage habitats. Front Microbiol 6:475. doi:10.3389/fmicb.2015.00475 26074887 PMC4448039

[75] Nordhoff M, Tominski C, Halama M, Byrne JM, Obst M, Kleindienst S, Behrens S, Kappler A. 2017. Insights into nitrate-reducing Fe(II) oxidation mechanisms through analysis of cell-mineral associations, cell encrustation, and mineralogy in the chemolithoautotrophic enrichment culture KS. Appl Environ Microbiol 83:1–19. doi:10.1128/AEM.00752-17 PMC5478975 28455336

[76] Silva PRA da, Simões-Araújo JL, Vidal MS, Cruz LM, Souza EM de, Baldani JI. 2018. Draft genome sequence of Paraburkholderia tropica Ppe8 strain, a sugarcane endophytic diazotrophic bacterium. Braz J Microbiol 49:210–211. doi:10.1016/j.bjm.2017.07.005 29122479 PMC5914139

[77] Jung M-Y, Kang M-S, Lee K-E, Lee E-Y, Park S-J. 2019. Paraburkholderia dokdonella sp. nov., isolated from a plant from the genus Campanula. J Microbiol 57:107–112. doi:10.1007/s12275-019-8500-5 30456756

[78] Sawana A, Adeolu M, Gupta RS. 2014. Molecular signatures and phylogenomic analysis of the genus Burkholderia: proposal for division of this genus into the emended genus Burkholderia containing pathogenic organisms and a new genus Paraburkholderia gen. nov. harboring environmental species. Front Genet 5:429. doi:10.3389/fgene.2014.00429 25566316 PMC4271702

[79] Pratama AA, Jiménez DJ, Chen Q, Bunk B, Spröer C, Overmann J, van Elsas JD. 2020. Delineation of a subgroup of the genus paraburkholderia, including P. terrae DSM 17804T, P. hospita DSM 17164T, and four soil-isolated fungiphiles, reveals remarkable genomic and ecological features-proposal for the definition of a P. hospita species cluster. Genome Biol Evol 12:325–344. doi:10.1093/gbe/evaa031 32068849 PMC7186790

[80] Aizawa T, Bao Ve N, Vijarnsorn P, Nakajima M, Sunairi M. 2010. Burkholderia acidipaludis sp. nov., aluminum-tolerant bacteria isolated from Chinese water chestnut (Eleocharis dulcis) growing in highly acidic swamps in South-East Asia. Int J Syst Evol Microbiol 60:2036–2041. doi:10.1099/ijs.0.018283-0 19819996

[81] Valverde A, Delvasto P, Peix A, Velázquez E, Santa-Regina I, Ballester A, Rodríguez-Barrueco C, García-Balboa C, Igual JM. 2006. Burkholderia ferrariae sp. nov., isolated from an iron ore in Brazil. Int J Syst Evol Microbiol 56:2421–2425. doi:10.1099/ijs.0.64498-0 17012573

[82] Otsuka Y, Muramatsu Y, Nakagawa Y, Matsuda M, Nakamura M, Murata H. 2011. Burkholderia oxyphila sp. nov., a bacterium isolated from acidic forest soil that catabolizes (+)-catechin and its putative aromatic derivatives. Int J Syst Evol Microbiol 61:249–254. doi:10.1099/ijs.0.017368-0 20207808

[83] Choi GM, Im WT. 2018. Paraburkholderia azotifigens sp. nov., a nitrogen-fixing bacterium isolated from paddy soil. Int J Syst Evol Microbiol 68:310–316. doi:10.1099/ijsem.0.002505 29185955

[84] Chen W-M, de Faria SM, Chou J-H, James EK, Elliott GN, Sprent JI, Bontemps C, Young JPW, Vandamme P. 2008. Burkholderia sabiae sp. nov., isolated from root nodules of Mimosa caesalpiniifolia. Int J Syst Evol Microbiol 58:2174–2179. doi:10.1099/ijs.0.65816-0 18768625

[85] Straub KL, Benz M, Schink B, Widdel F. 1996. Anaerobic, nitrate-dependent microbial oxidation of ferrous iron. Appl Environ Microbiol 62:1458–1460. doi:10.1128/aem.62.4.1458-1460.1996 16535298 PMC1388836

[86] Valkanas MM, Rosso T, Packard JE, Trun NJ. 2021. Limited carbon sources prevent sulfate remediation in circumneutral abandoned mine drainage. FEMS Microbiol Ecol 97:fiaa262. doi:10.1093/femsec/fiaa262 33417684

