# Supplementary material for: Bacterial nitrite production oxidizes Fe(II) bioremediating acidic abandoned coal mine drainage
Source: Appl Environ Microbiol. 2025 Apr 16;91(5):e00405-25. doi: 10.1128/aem.00405-25 (PMC12094003; doi:10.1128/aem.00405-25)
Supplement: Supplemental material — Tables A1 to A4; Fig. A1 and A2. [file aem.00405-25-s0001.pdf]

APPENDIXES

Table A1. Development of Synthetic Abandoned Mine Drainage (AMD). Synthetic AMD was created to control for seasonal changes between samplings. The seasonal levels of lithium, boron, titanium, vanadium, chromium, cobalt, nickel, copper, zinc, arsenic, rubidium, strontium, molybdenum, cadmium, antimony, barium, tungsten, lead, uranium, selenium, sulfate, sodium, magnesium, aluminum, phosphorus, potassium, calcium, manganese, iron, and silicon were determined with ion chromatography (**IC**) and inductively coupled plasma mass spectrometry (**ICP-MS**) [1]. The seasonal average, high, low, and standard deviation of each contaminant was determined for the system. Laboratory chemical compounds were used to mimic the chemical conditions frequently found at Boyce Park by determining the molarity of each chemical using its molecular weight and pH adjusted to 4.0 with sulfuric acid. Chemicals used to develop synthetic AMD include lithium sulfate (Li_2SO_4), sodium borate ($Na_2B_4O_7 \bullet 10H_2O$), vanadyl sulfate ($VOSO_4$), potassium chromate ($K_2CrO_4$), cobalt chloride ($CoCl_2 \bullet 6H_2O$), nickel chloride ($NiCl_2$), copper sulfate pentahydrate ($CuSO_4 \bullet 5H_2O$), zinc sulfate heptahydrate ($ZnSO_4 \bullet 7H_2O$), sodium arsenite ($NaAsO_2$), strontium chloride hexahydrate ($Cl_2Sr \bullet 6H_2O$), sodium molybdate ($Na_2MoO_4 \bullet 2H_2O$), barium chloride ($BaCl_2 \bullet 2H_2O$), sodium tungstate ($Na_2WO_4 \bullet 2H_2O$), lead nitrate ($Pb(NO_3)_2$), sodium selenite (Na_2SeO_3), sodium sulfate ($Na_2SO_4 \bullet 10H_2O$), magnesium sulfate ($MgSO_4 \bullet 7H_2O$), aluminum potassium sulfate dodecahydrate ($KAl(SO_4)_2 \bullet 12H_2O$), aluminum sulfate ($Al_2(SO_4)_3 \bullet 18H_2O$), potassium phosphate ($K_2HPO_4 \bullet 3H_2O$), potassium sulfate ($K_2SO_4$), calcium nitrate ($Ca(NO_3)_2 \bullet 4H_2O$), calcium chloride ($CaCl_2 \bullet 2H_2O$), calcium sulfate ($CaSO_4 \bullet 2H_2O$), manganese sulfate monohydrate ($MnSO_4 \bullet H_2O$), ferrous sulfate heptahydrate ($FeSO_4 \bullet 7H_2O$), silicon dioxide (SiO_2). Synthetic AMD measurements highlighted in yellow are within 10-fold of the seasonal average, those highlighted in blue are within 5-fold, and those in green are in 2-fold. Number reported for readability.

	PPM				
**Chemical Contaminant**	**EPA Limits**	**Boyce AMD** Avg ± SD	High	Low	**Synthetic AMD**
**Chloride**	250	25.6 ± 39.4	84.605	3.906	4.8
**Nitrate**	44.3	1.588 ± 1.312	2.516	bdl	bdl
**Sulfate**	250	760 ± 158	909.536	616.528	90.6
**Lithium**	NA	0.241 ± 0.040	0.292	0.2	0.0276
**Boron**	NA	0.033 ± 0.008	0.043	0.024	0.0215
**Sodium**	NA	15.411 ± 15.587	38.657	5.845	7.93
**Magnesium**	NA	34.857 ± 9.801	46.077	25.391	2.14
**Aluminum**	0.05	70.0 ± 29.2	107.48	36.849	12.2945
**Silicon**	NA	13.22 ± 4.48	17.811	7.235	bdl
**Phosphorus**	NA	0.018 ± NA	0.018	bdl	bdl
**Potassium**	NA	0.374 ± 0.250	0.593	0.069	0.3416
**Calcium**	NA	84.2 ± 16.5	103.847	68.75	2.2196
**Vanadium**	NA	0.0027 ± 0.0030	0.005	<0.001	0.0006
**Chromium**	0.1	0.0088 ± 0.0047	0.015	0.004	0.0025
**Manganese**	0.05	0.427 ± 0.084	0.522	0.317	0.0615
**Iron**	0.3	4.258 ± 0.263	4.489	3.959	0.744
**Cobalt**	NA	0.086 ± 0.031	0.122	0.047	0.0211
**Nickel**	NA	0.260 ± 0.044	0.315	0.21	0.011
**Copper**	<1.3	0.107 ± 0.019	0.126	0.084	0.2323
**Zinc**	5	0.348 ± 0.051	0.416	0.295	0.0932
**Arsenic**	0.01	0.0014 ± 0.0011	0.002	<0.001	0.001
**Selenium**	0.05	0.0035 ± 0.0015	0.005	bdl	0.0009
**Strontium**	NA	0.156 ± 0.050	0.208	0.111	0.0278
**Molybdenum**	NA	0.000127 ± 0.000067	0	0	0.0005
**Barium**	2	0.013 ± 0.002	0.014	<0.01	0.0001
**Tungsten**	NA	0.007 ± 0.003	0.01	0.004	0.004
**Lead**	0.015	0.0016 ± 0.0010	0.003	0.001	0.0005

Parts per million (**PPM**), Environmental protection agency (**EPA**), Abandoned mine drainage (**AMD**), Standard deviation (**SD**), Below detectable limit (**bdl**), Not applicable (**NA**)

Table A2. Novel forward and reverse primers designed for *Paraburkholderia* sp. AV18 to target their *napA* and *rpoB genes* for PCR and gene expression.

Gene	Primer	Sequence	Primer Length (bp)	Hairpin (ΔG)	Self-Dimer (ΔG)
napA	napA_F1	5' AAGAGCGTAAAGAGCGTGTGTCC 3'	23	-0.98	-3.61
napA	napA_R1	5' CGTGCTTGTTGACGAGATACTGCG 3'	24	-0.66	-4.95
rpoB	rpoB_F1	5' CCCATCGTTCACCAGGTTCC 3'	20	0.56	-4.41
rpoB	rpoB_R1	5' ATTCCTTGATGTTGAATGCCG 3'	21	0.15	-4.99

Table A3. Novel primer sets designed for *Paraburkholderia* sp. AV18 to target their *napA* and *rpoB genes* for PCR and gene expression.

Gene	Forward	Reverse	Hetero-Dimer (ΔG)	Expected PCR Product Size (bp)
napA	napA_F1	napA_R1	-4.92	352
rpoB	rpoB_F1	rpoB_R1	-5.47	208

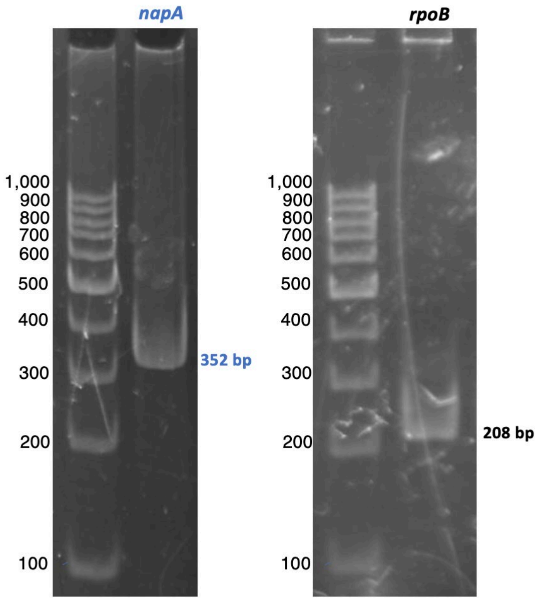

Fig A1. Validation of novel primer sets for *napA* and *rpoB* from AV18 with DNA. Lane 1 is GeneRuler 100 bp DNA Ladder (Thermo Fisher Scientific, Waltham, MA, USA). Lane 2 is novel primer set. Novel primer set for *napA* was expected to be 352 bp and the band is at that location and for *rpoB* was expected to be 208 bp and the band is at that location.

Table A4. Total ASVs for Boyce Park PRS and Middle Branch PRS for Each Location Sampled.

PRS	Location	Individual ASVs
Boyce	Pond 1	774
Boyce	Pond 2	779
Boyce	Pond 3	1,063
Boyce	Pond 4	926
Boyce	Pond 5	680
Boyce	Pond 6	1,179
Boyce	Pond 7	1,073
Boyce	Pond 8	1,196
Middle Branch	Pond MB1	588
Middle Branch	Pond MB2	1,119
Middle Branch	Pond MB3	821
Middle Branch	Pond MB4	1,234
Middle Branch	Pond MB5	960
Middle Branch	Pond MB6	1,232
Middle Branch	Pond MB7	1,459

Passive remediation system (PRS)
Individual amplicon sequence variants (ASVs)

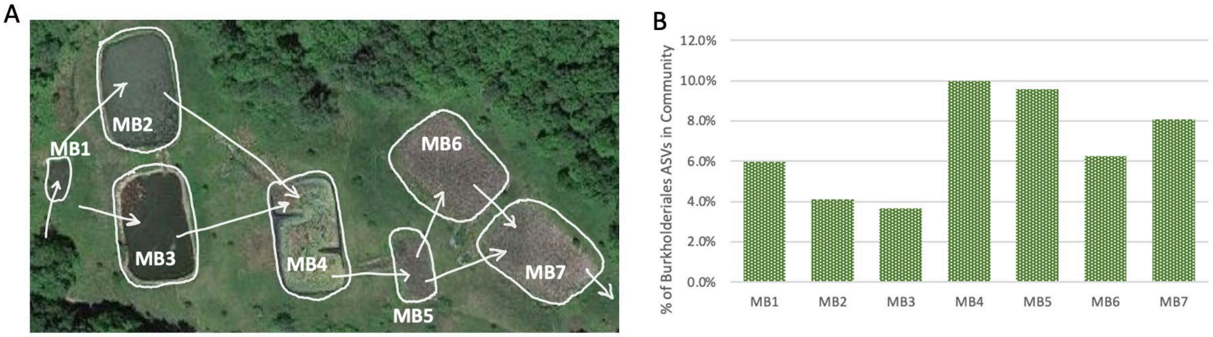

Fig A2. Acidic Middle Branch AMD passive remediation system (PRS). A. Aerial image of Middle Branch PRS with pond numbering and AMD flow within the system updated from Valkanas 2020 [2]. Map data ©2024 Mapbox ©OpenStreetMap ©Maxar. MB1 and MB4 are settling ponds, MB2, MB3, MB6, and MB7 are vertical flow ponds, and lastly, MB5 is a wetland. Flow of AMD through the Middle Branch PRS is shown by the arrows. **B. Middle Branch PRS 16S rRNA gene sequencing of the mixed microbial community.** Burkholderiales are present within every pond at the acidic Middle Branch PRS as determined by 16S rRNA gene community sequencing.

1. Cantlay, T., et al., *Determining conventional and unconventional oil and gas well brines in natural sample II: Cation analyses with ICP-MS and ICP-OES.* J Environ Sci Health A Tox Hazard Subst Environ Eng, 2020. **55**(1): p. 11-23.
2. Valkanas, M., *Identifying the Effects Naturally Forming Bacterial Communities Have on the Efficiency of Passive Remediation Systems Built to Treat Abandoned Coal Mine Drainage*, in *Biological Sciences*. 2020, Duquesne University. p. 532.
